# Supplementary material for: Synthesis and characterization of novel ssDNA X-aptamers targeting Growth Hormone Releasing Hormone (GHRH)
Source: PLoS One. 2022 Jan 21;17(1):e0260144. doi: 10.1371/journal.pone.0260144 (PMC8782341; doi:10.1371/journal.pone.0260144)
Supplement: S1 Raw images — (PDF) [file pone.0260144.s001.pdf]

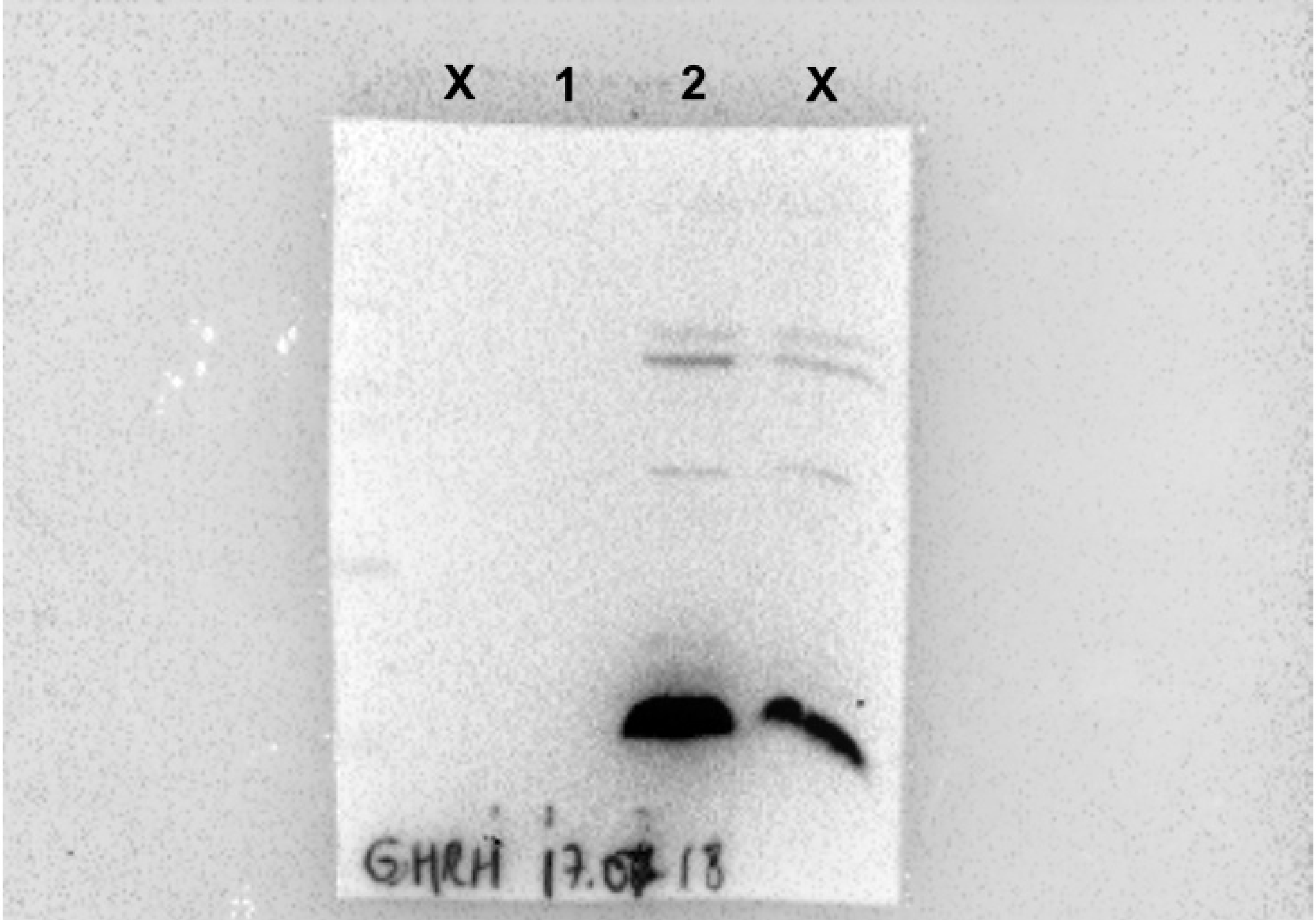

Figure 1a, upper panel. (1) E. coli HB101 His vector clone (2) E. coli HB101 His-GHRH vector clone. The membrane image was captured by ChemiDoc MP Imaging System after Chemiluminescence Staining

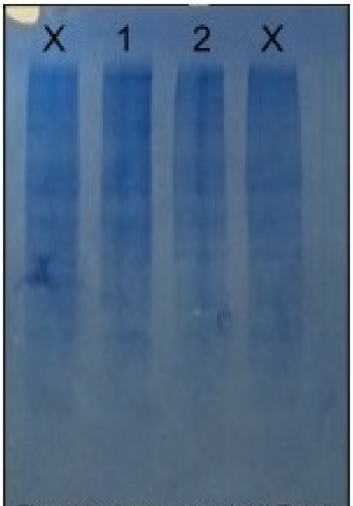

Figure 1a, bottom panel. (1) E. coli HB101 His vector clone (2) E. coli HB101 His-GHRH vector clone. The gel was stained with Coomassie Brilliant Blue G-250 after transfer to membrane. Image was captured under white light by ChemiDoc MP Imaging Systen

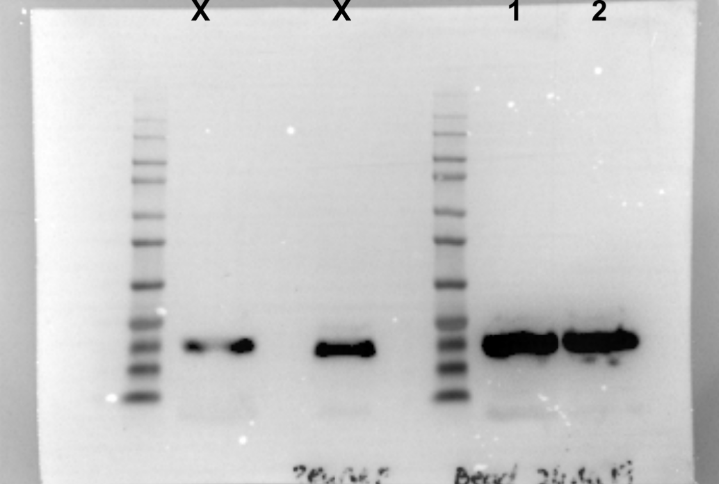

Figure 1b, upper panel, GHRH. (1) His-GHRH expressing total bacterial lysate (2) His-GHRH expressing solubilized pellets. The membrane image was captured by ChemiDoc MP Imaging System

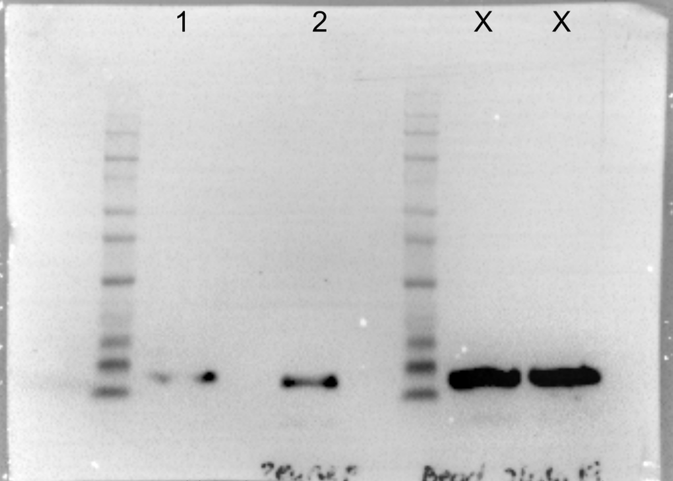

Figure 1b, upper panel, His (1) His-GHRH expressing total bacterial lysate (2) His-GHRH expressing solubilized pellets. The membrane was captured by ChemiDoc MP Imaging System after Chemiluminescence staining

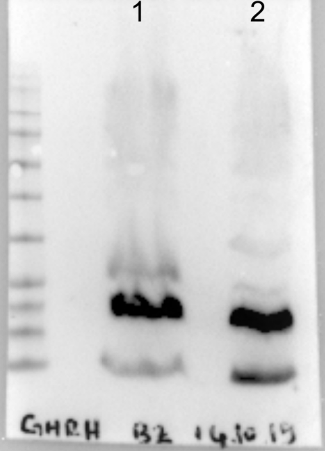

Figure 1b, bottom panel, GHRH (1) His-GHRH expressing total bacterial lysate (2) His-GHRH expressing solubilized pellets. The membrane was captured by ChemiDoc MP Imaging System after Chemiluminescence staining

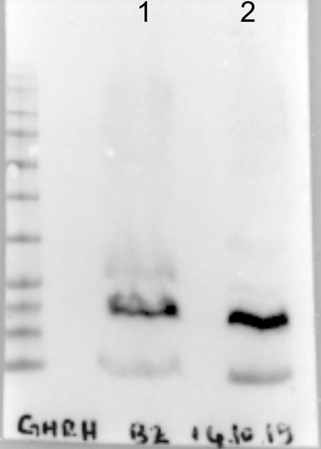

Figure 1b, bottom panel, His (1) His-GHRH expressing total bacterial lysate (2) His-GHRH expressing solubilized pellets. The membrane was captured by ChemiDoc MP Imaging System after Chemiluminescence staining

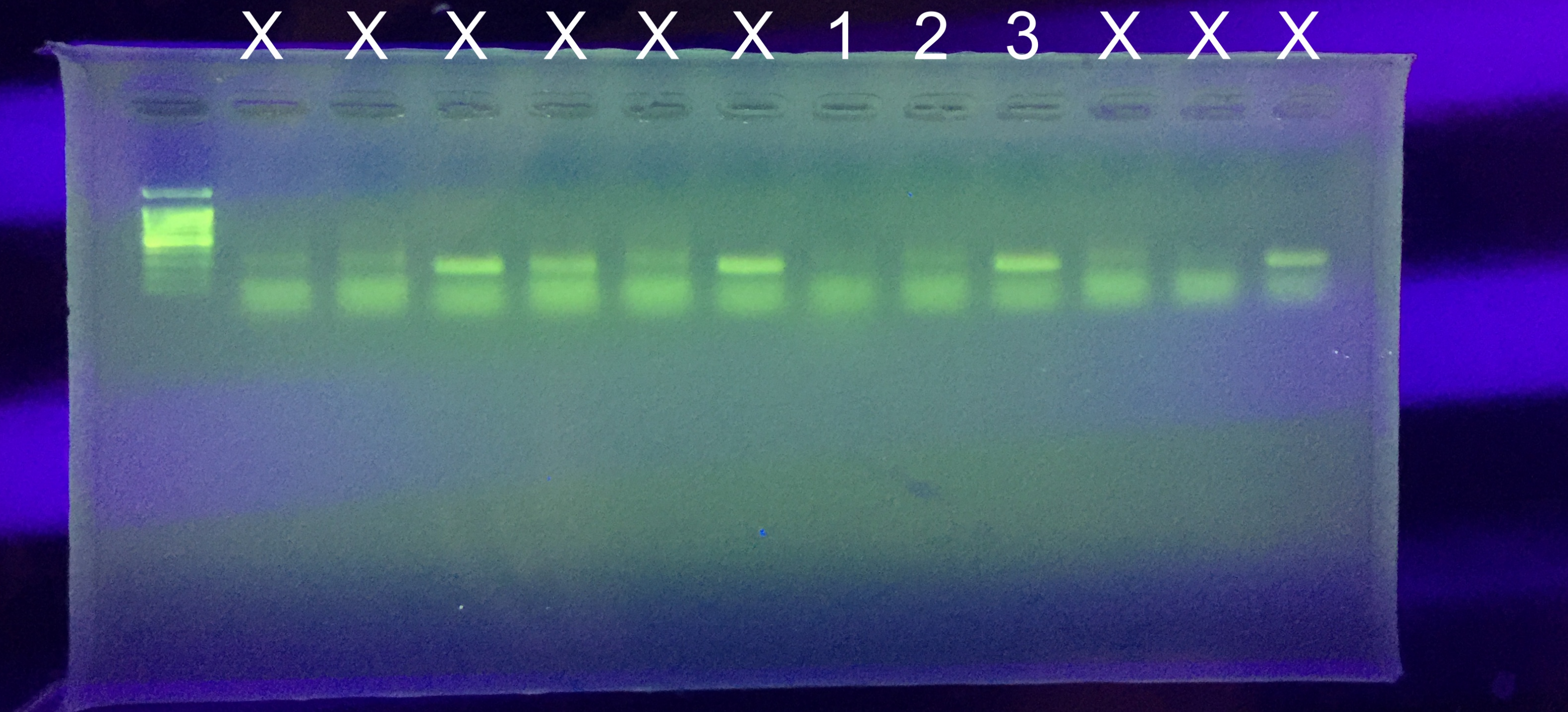

Figure 1c, GHRH (1) HEK293 wt (2) HEK293 His-transfected (3) HEK293 His-GHRH transfected. Agarose gel was visualized under UV light

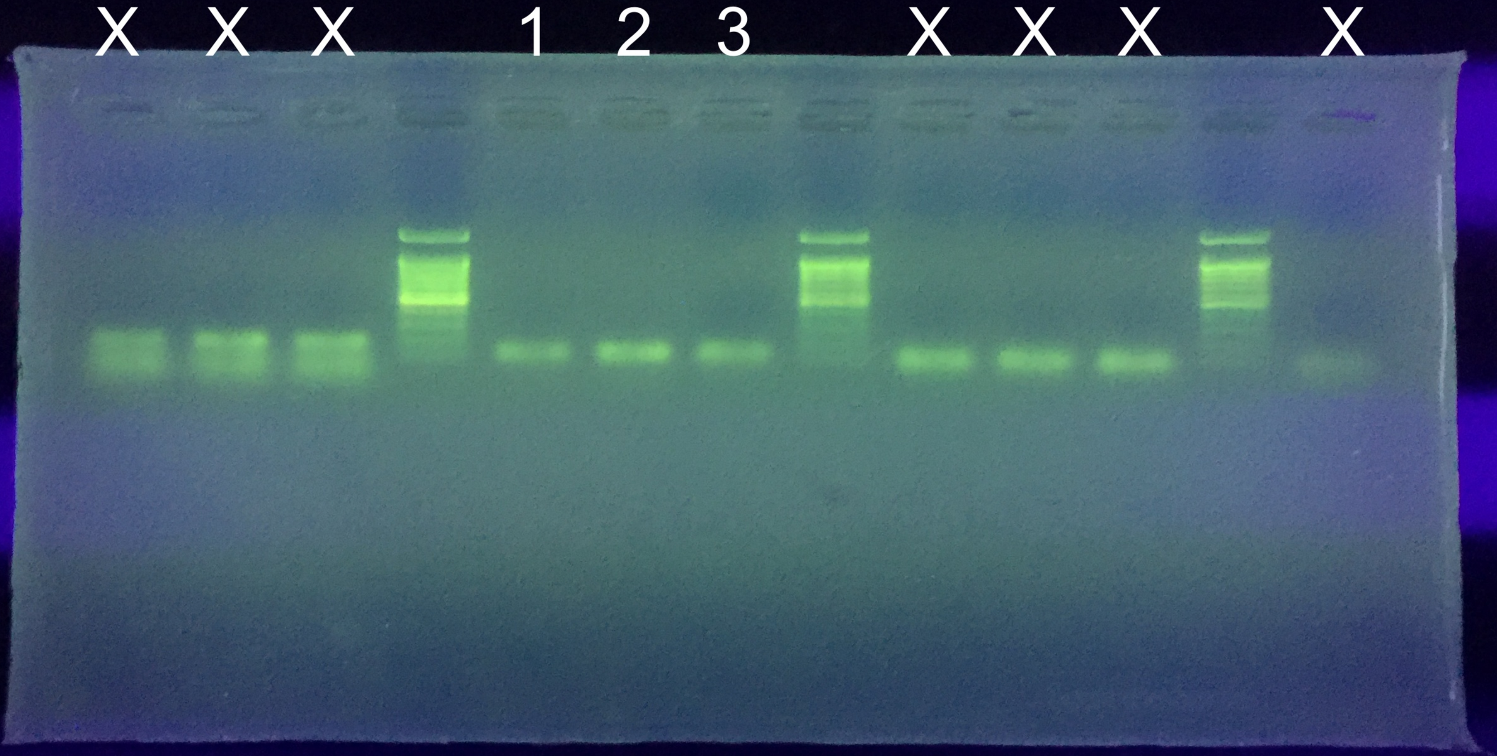

Figure 1c, 18S (1) HEK293 wt (2) HEK293 His-transfected (3) HEK293 His-GHRH transfected. Agarose gel was visualized under UV light

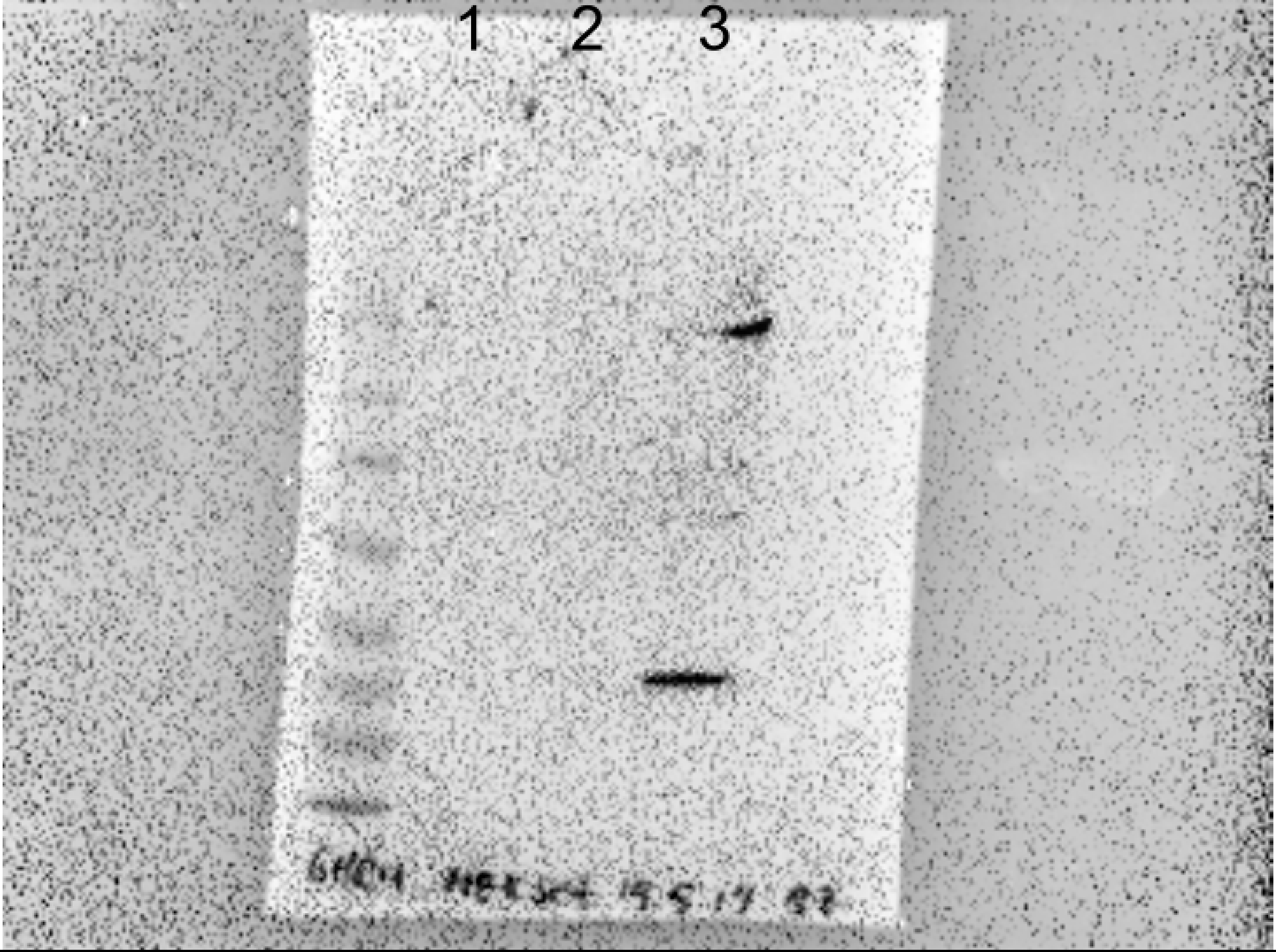
1
2
3

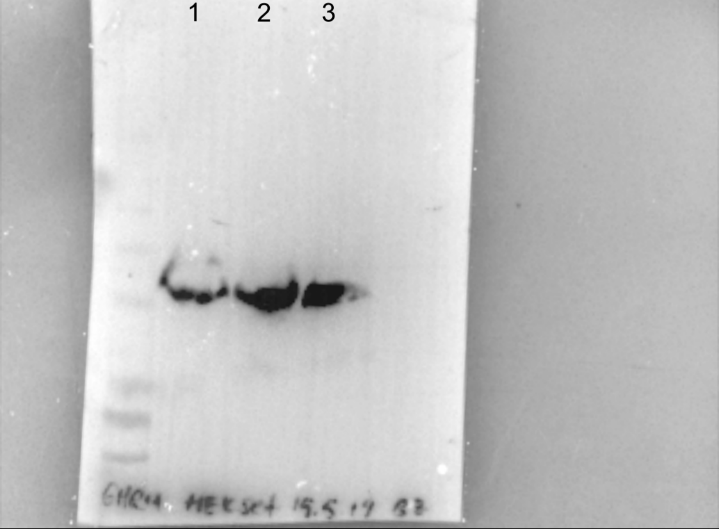
1
2
3

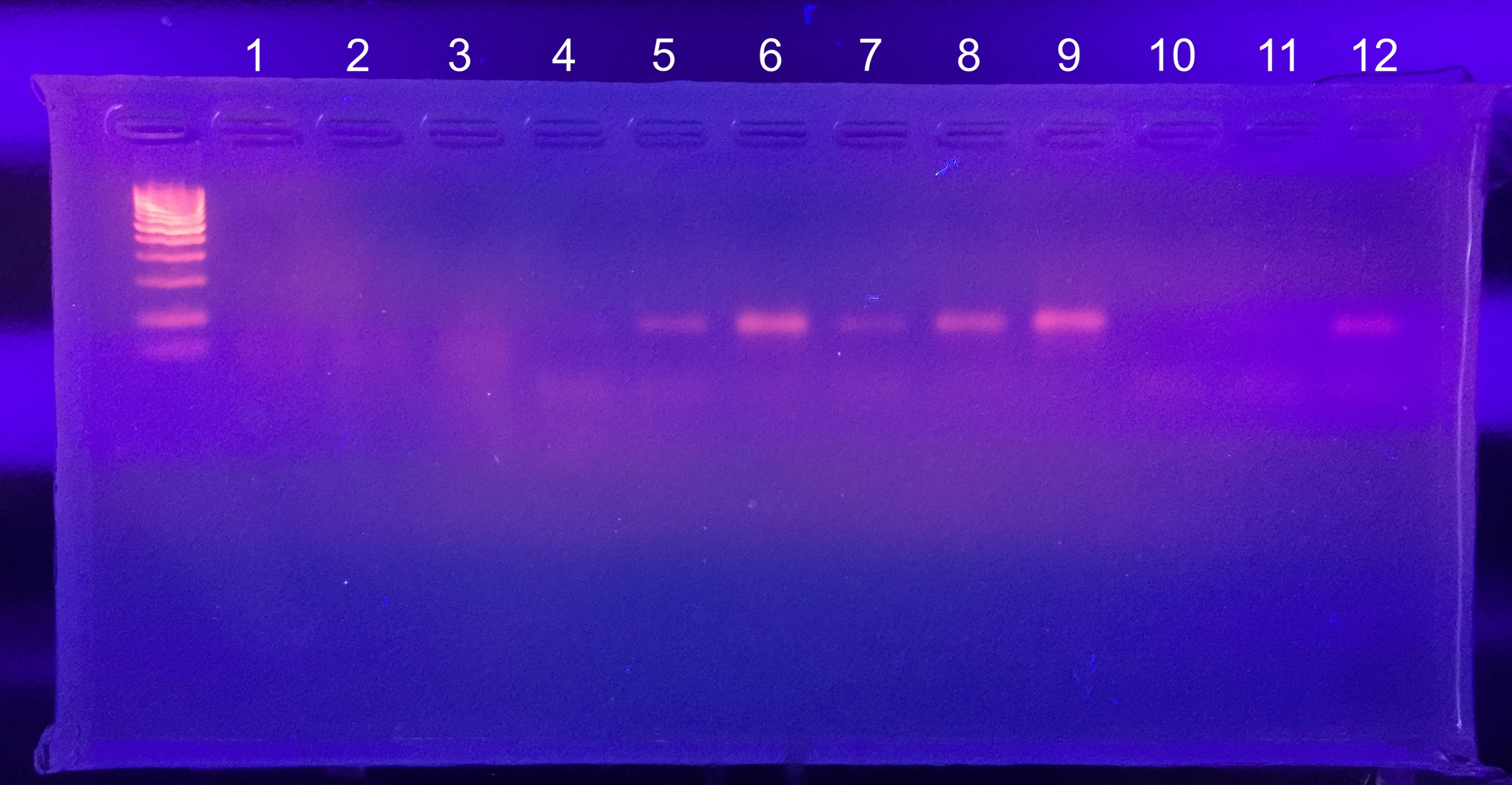

Figure 2a, upper panel. (1) Tube #1 14 cycle (2) Tube #1 18 cycle (3) Tube #1 22 cycle (4) Tube #2 14 cycle (5) Tube #2 18 cycle (6) Tube #2 22 cycle (7) Tube #3 14 cycle (8) Tube #3 18 cycle (9) Tube #3 22 cycle (10) Tube #7 14 cycle (11) Tube #7 18 cycle (12) Tube #7 22 cycle. Agarose gel was visualized under UV light

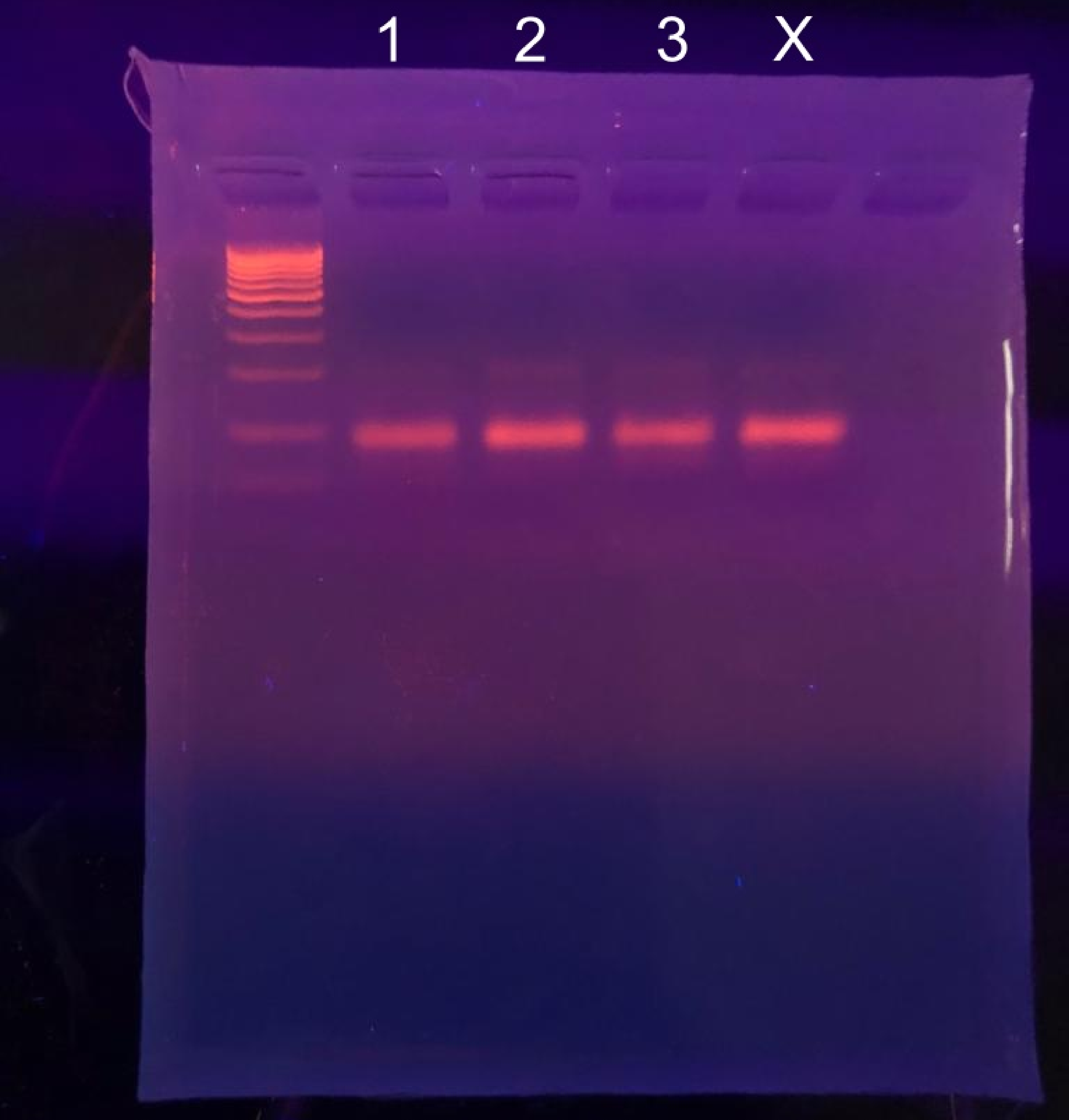

Figure 2a, bottom panel. (1) Tube #1: 25 cycle (2) Tube #2: 25 cycle (3) Tube #7: 25 cycle. Agarose gel was visualized under UV light

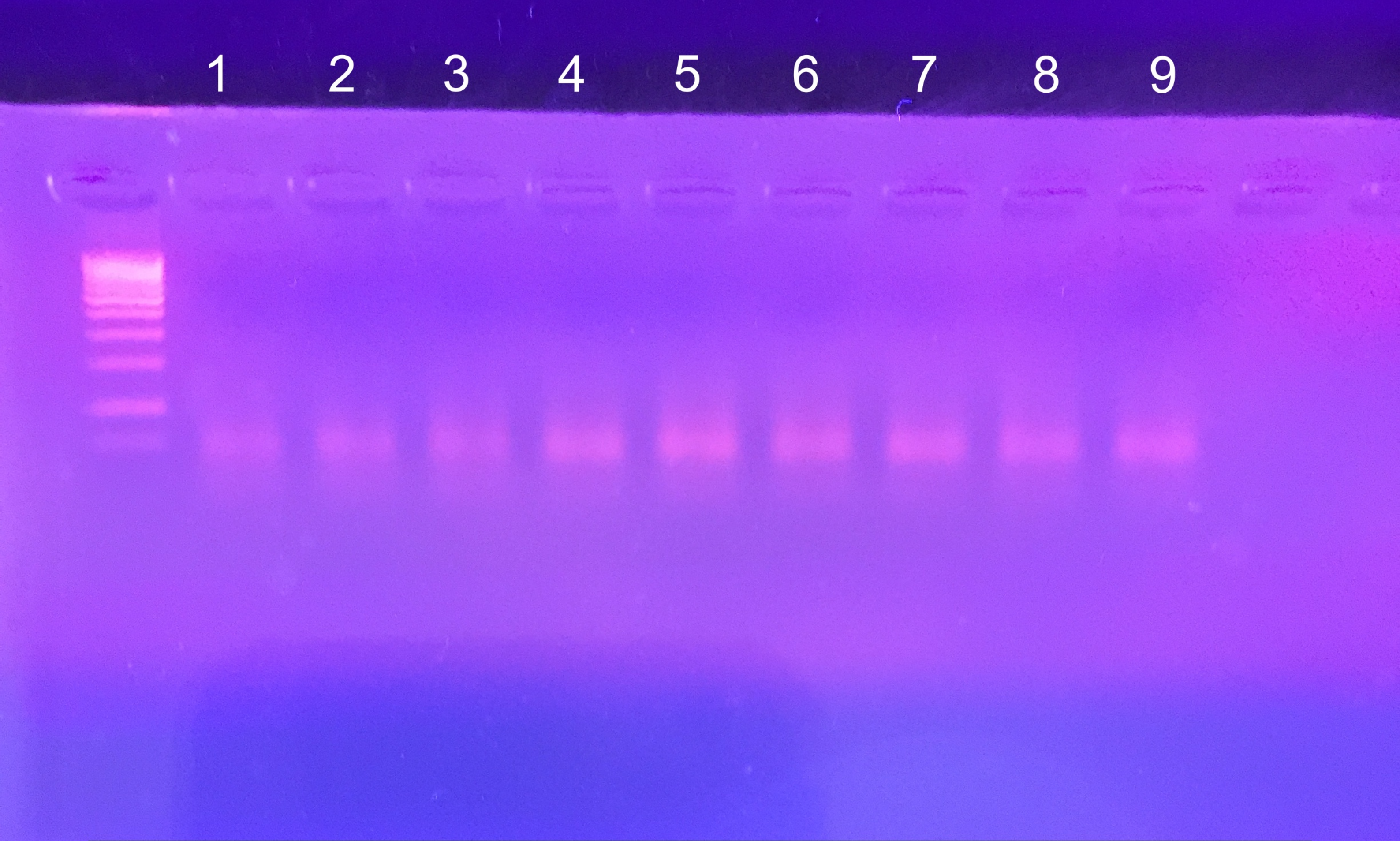

Figure 2b, upper panel. (1) Tube #1 14 cycle (2) Tube #1 18 cycle (3) Tube #1 22 cycle (4) Tube #2 14 cycle (5) Tube #2 18 cycle (6) Tube #2 22 cycle (7) Tube #7 14 cycle (8) Tube #7 18 cycle (9) Tube #7 22 cycle. Agarose gel was visualized under UV light

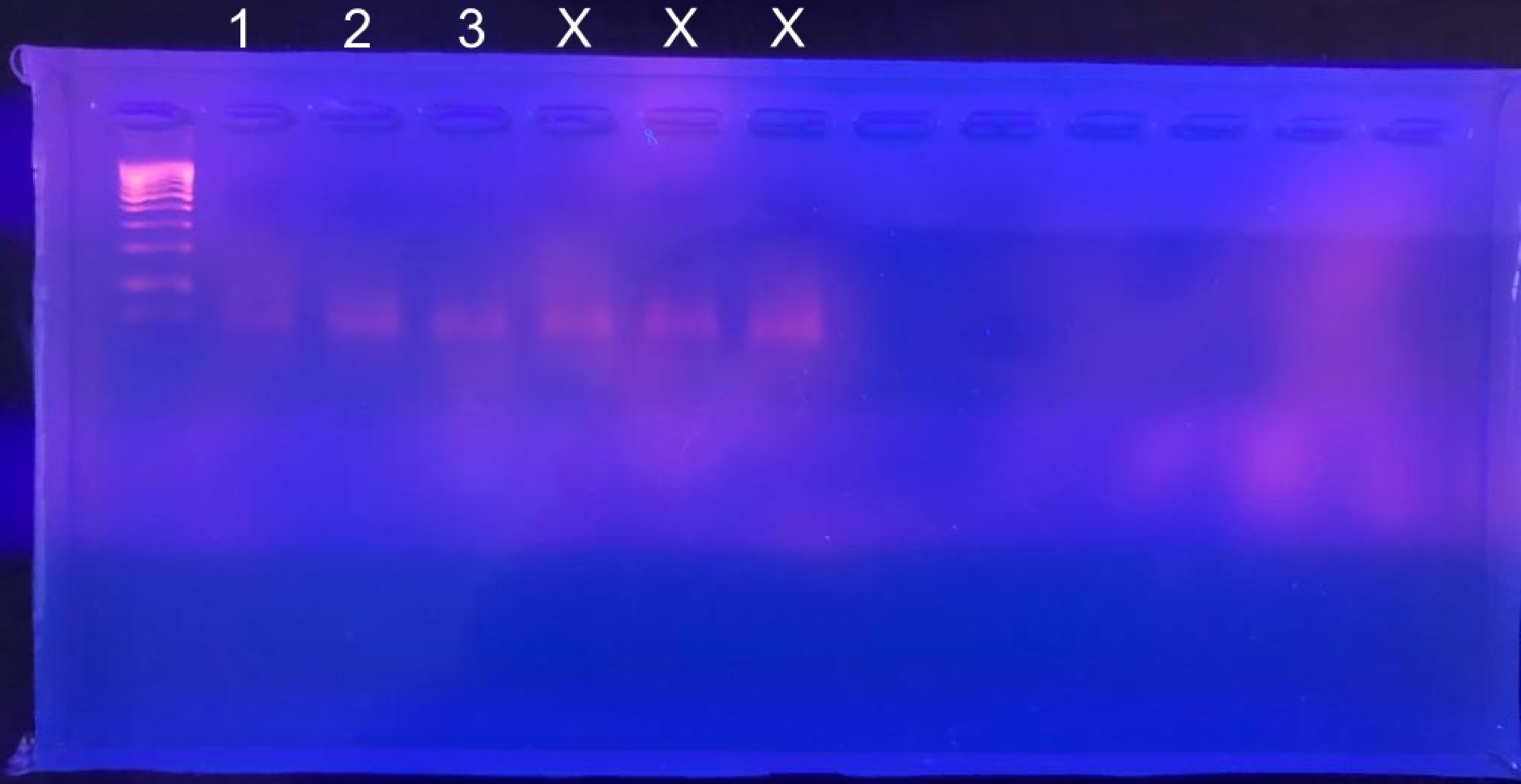

Figure 2b, bottom panel. (1) Tube #1 25 cycle (2) Tube #2 25 cycle (3) Tube #7 25 cycle. Agarose gel was visualized under UV light

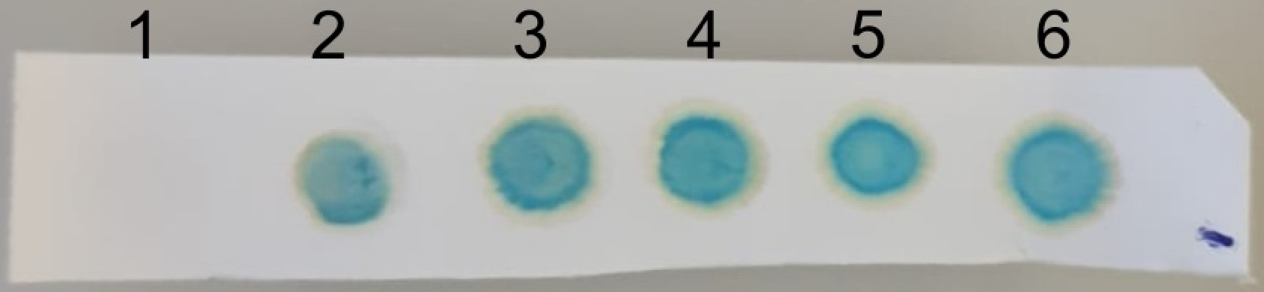

Figure 3a Lane 1. (1) Negative Control (2) TKY2.T1.01 (3) TKY2.T1.02 (4) TKY2.T1.03 (5) TKY2.T1.04 (6) TKY2.T1.05. HRP-treated membrane was incubated with TMB substrate and image was captured by LCD Camera

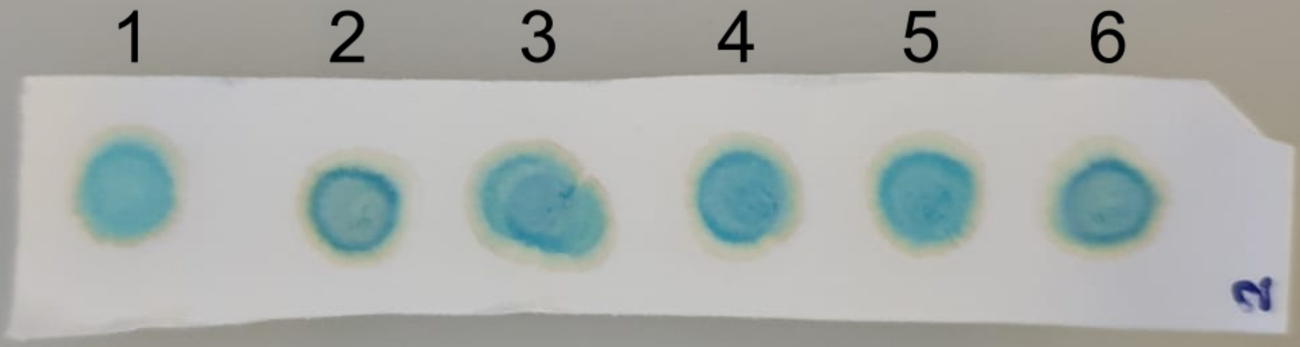

Figure 3a Lane 2. (1) TKY2.T1.06 (2) TKY2.T1.08 (3) TKY2.T1.10 (4) TKY2.T1.11 (5) TKY2.T1.12 (6) TKY2.T1.13. HRP-treated membrane was incubated with TMB substrate and image was captured by LCD Camera

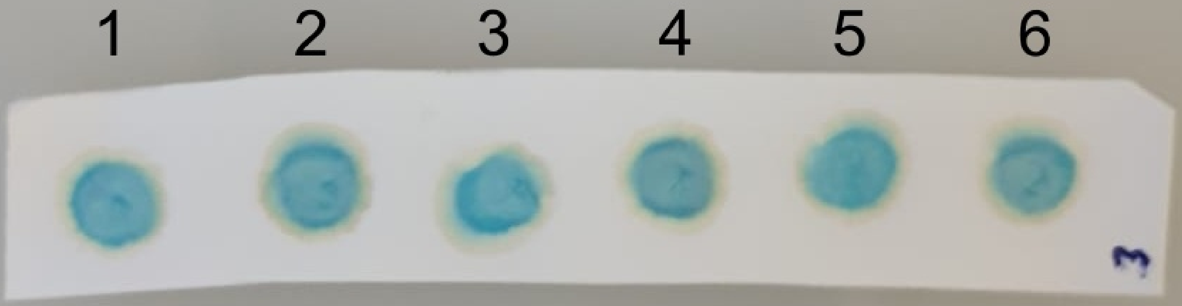

Figure 3a Lane 3. (1) TKY2.T1.15 (2) TKY2.T1.16 (3) TKY2.T1.17
(4) TKY.T1.01 (5) TKY.T1.02 (6) TKY.T1.03. HRP-treated membrane
was incubated with TMB substrate and image was captured by LCD Camera

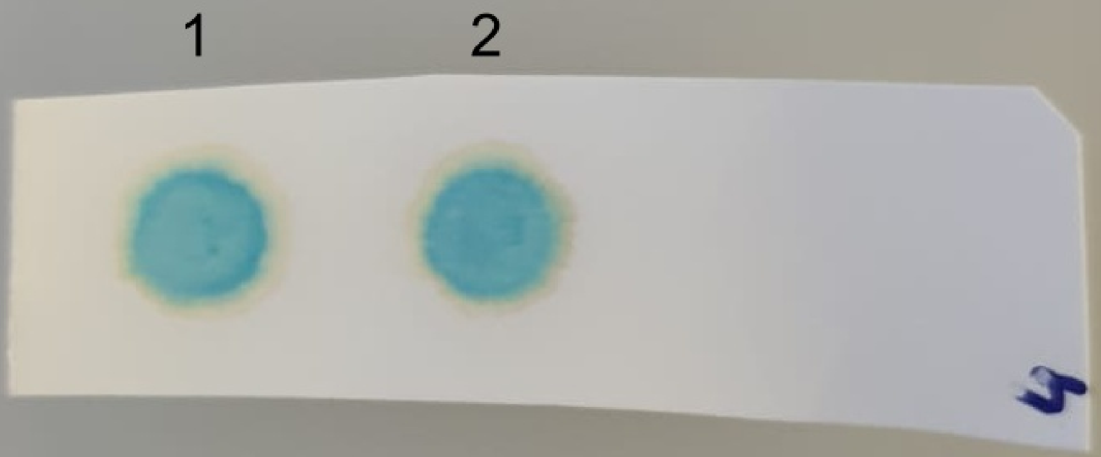

Figure 3a Lane 1. (1) TKY.T1.04 (2) TKY.T1.05. HRP-treated membrane was incubated with TMB substrate and image was captured by LCD Camera

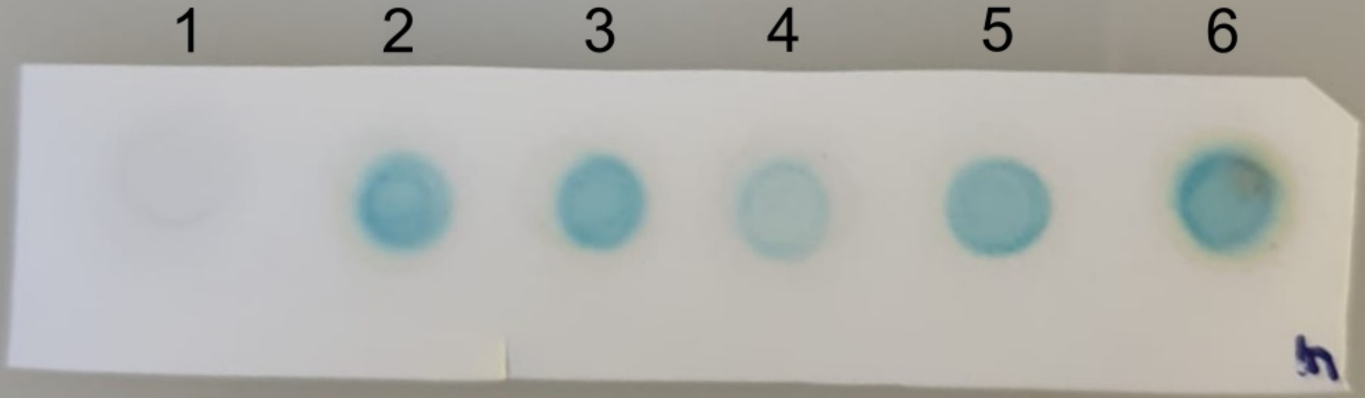

Figure 3b. (1) Negative Control (2) TKY.T2.02 (3) TKY.T2.06 (4) TKY.T2.07 (5) TKY.T2.08 (6) TKY.T2.09. HRP-treated membrane was incubated with TMB substrate and image was captured by LCD Camera

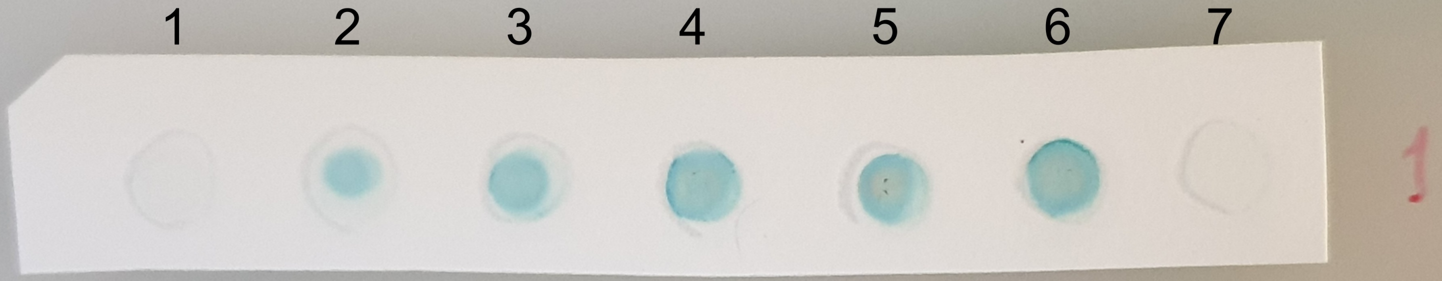

Figure 3c TKY2.T1.01. (1) 0 nM (2) 50 nM (3) 100 nM (4) 250 nM (5) 500 nM (6) 1000 nM (7) Scrambled aptamer. HRP-treated membrane was incubated with TMB substrate and image was captured by LCD Camera

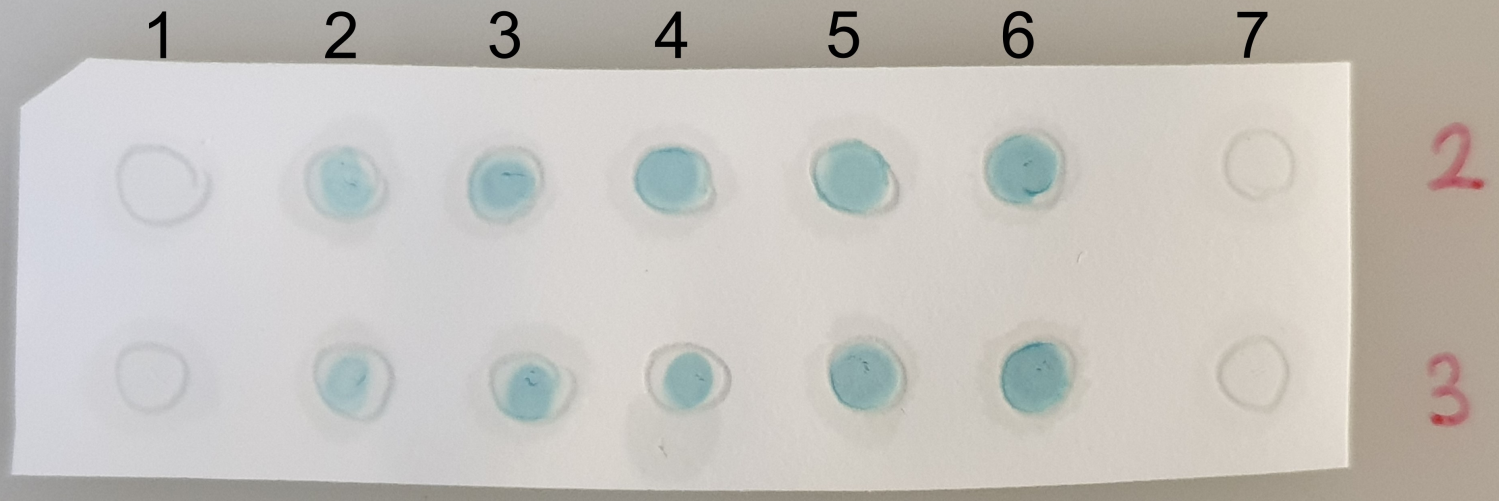

Figure 3c, TKY2.T1.02-upper lane, TKY2.T1.03-bottom lane. (1) 0 nM (2) 50 nM (3) 100 nM (4) 250 nM (5) 500 nM (6) 1000 nM (7) Scrambled aptamer. HRP-treated membrane was incubated with TMB substrate and image was captured by LCD Camera

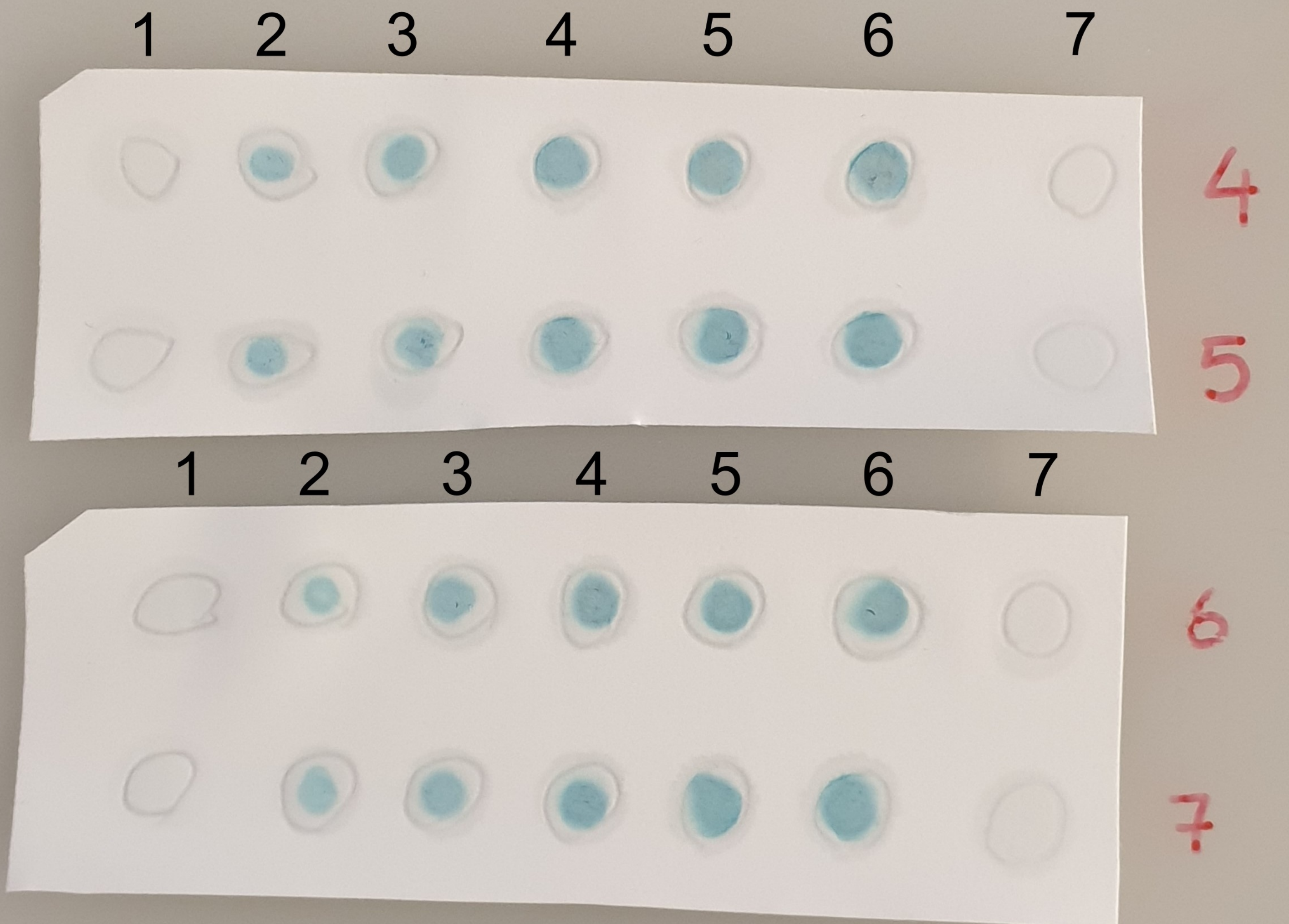

Figure 3c, TKY2.T1.04-upper membrane first lane, TKY2.T1.05-upper membrane second lane, TKY2.T1.06-lower membrane first lane, TKY2.T1.08-lower membrane second lane. (1) 0 nM (2) 50 nM (3) 100 nM (4) 250 nM (5) 500 nM (6) 1000 nM (7) Scrambled aptamer. HRP-treated membrane was incubated with TMB substrate and image was captured by LCD Camera

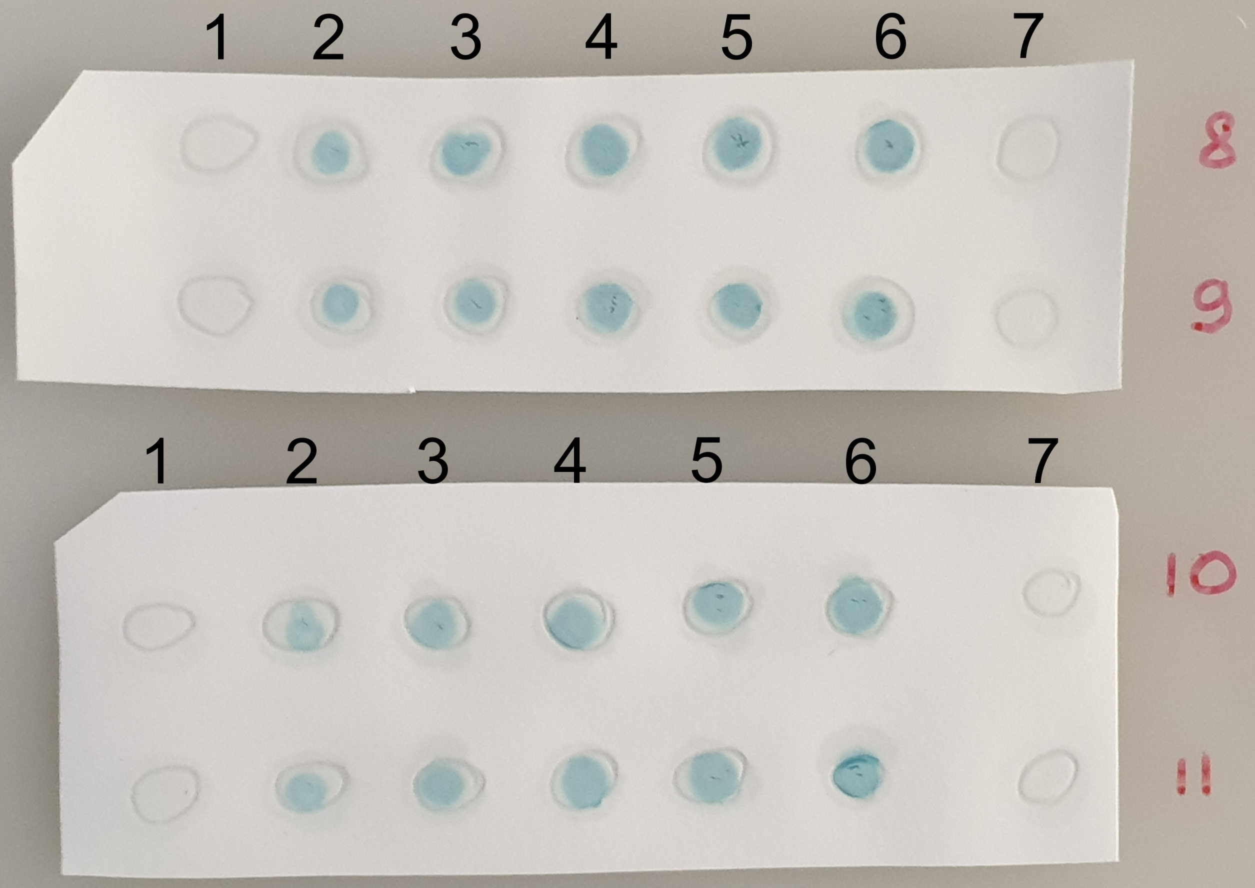

Figure 3c, TKY2.T1.10-upper membrane first lane, TKY2.T1.11-upper membrane second lane, TKY2.T1.12-lower membrane first lane, TKY2.T1.13-lower membrane second lane. (1) 0 nM (2) 50 nM (3) 100 nM (4) 250 nM (5) 500 nM (6) 1000 nM (7) Scrambled aptamer. HRP-treated membrane was incubated with TMB substrate and image was captured by LCD Camera

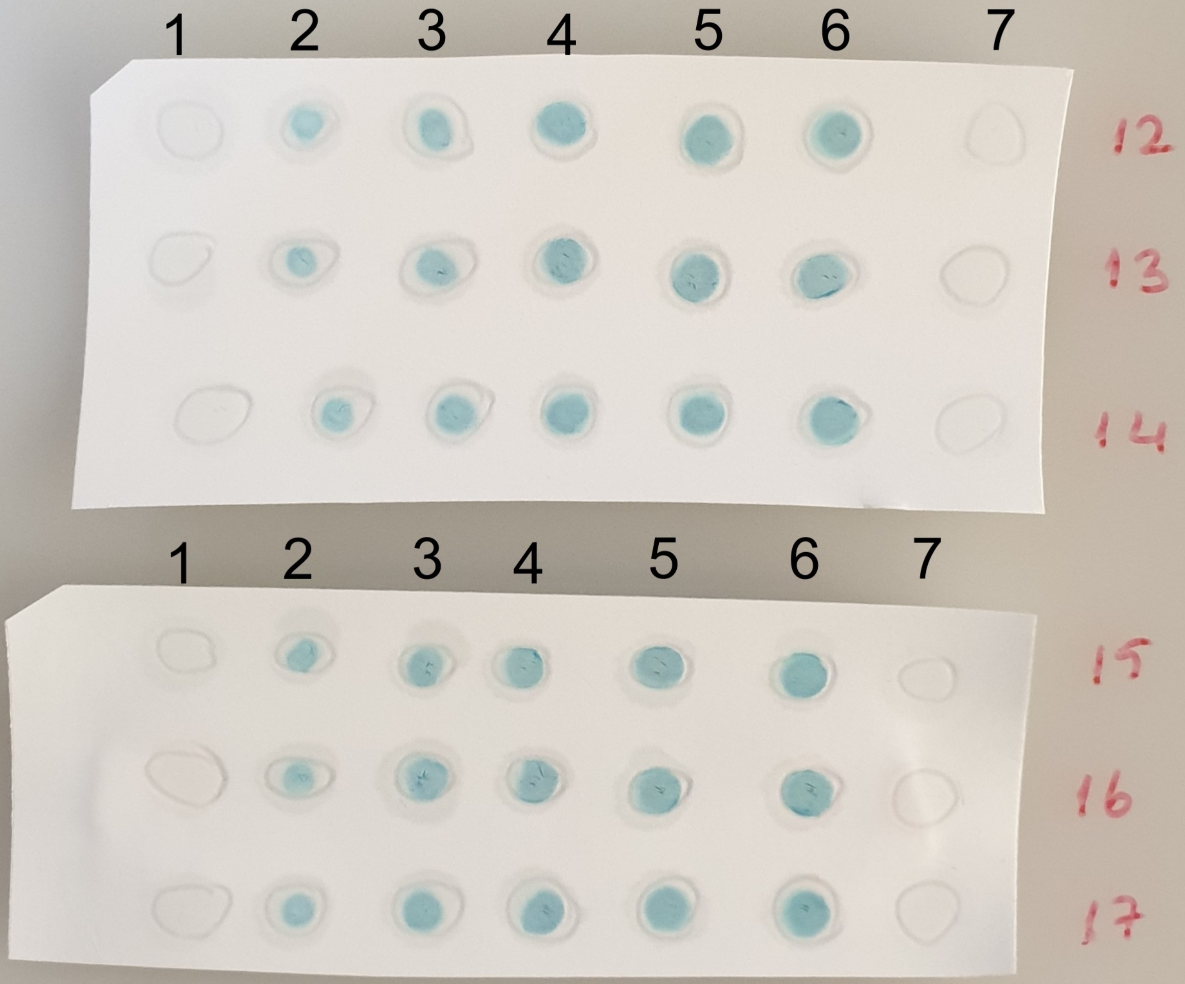

Figure 3c, TKY2.T1.15-upper membrane first lane, TKY2.T1.16-upper membrane second lane, TKY2.T1.17-upper membrane third lane, TKY.T1.01-lower membrane first lane, TKY.T1.02-lower membrane second lane, TKY.T1.03-lower membrane third lane. (1) 0 nM (2) 50 nM (3) 100 nM (4) 250 nM (5) 500 nM (6) 1000 nM (7) Scrambled aptamer. HRP-treated membrane was incubated with TMB substrate and image was captured by LCD Camera

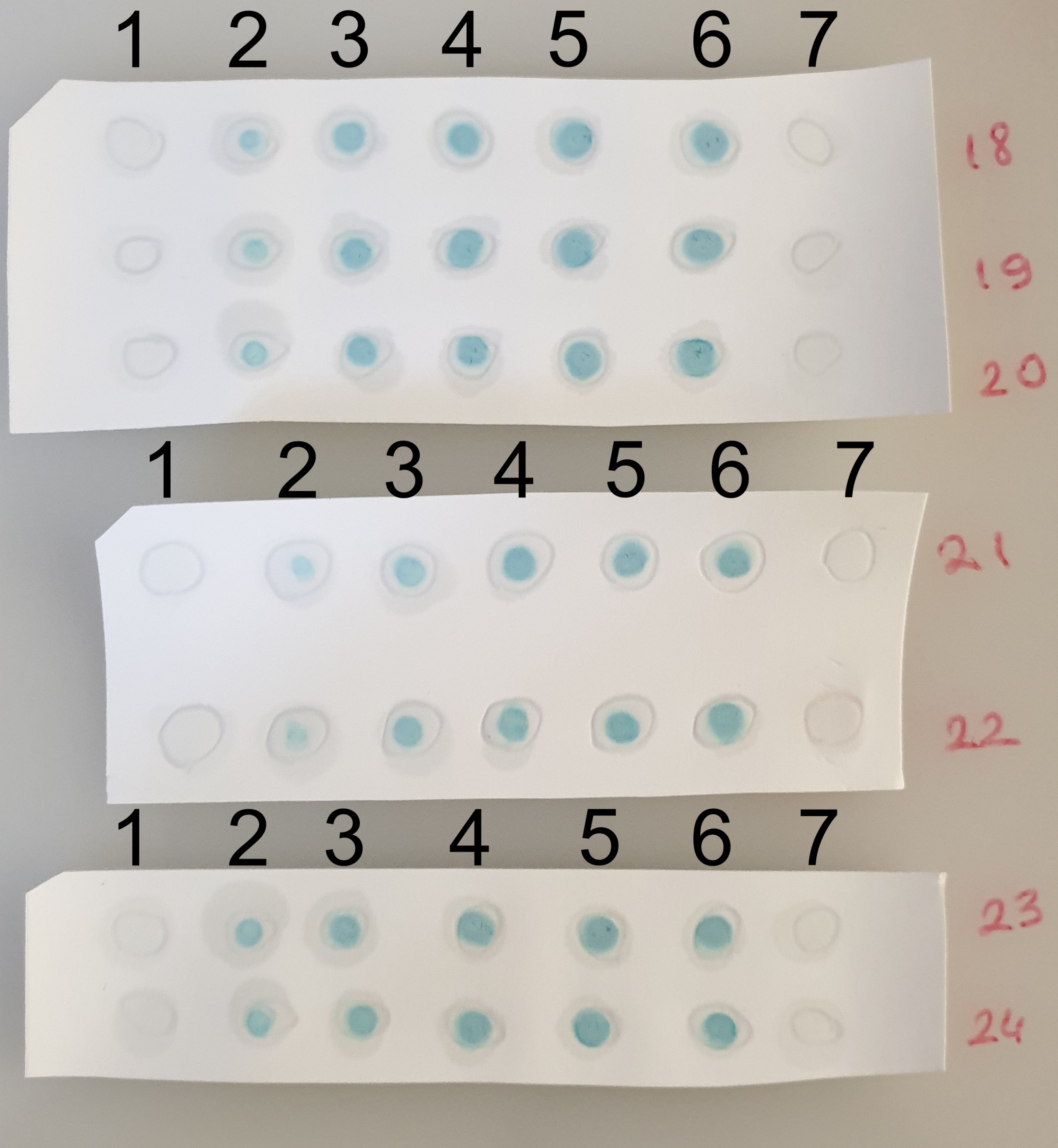

Figure 3c, TKY.T1.04-upper membrane first lane, TKY.T1.05-upper membrane second lane, TKY.T2.02-upper membrane third lane, TKY.T2.06-middle membrane first lane, TKY.T2.07-middle membrane second lane, TKY.T2.08-lower membrane first lane, TKY.T2.09-lower membrane second lane (1) 0 nM (2) 50 nM (3) 100 nM (4) 250 nM (5) 500 nM (6) 1000 nM (7) Scrambled aptamer. HRP-treated membrane was incubated with TMB substrate and image was captured by LCD Camera

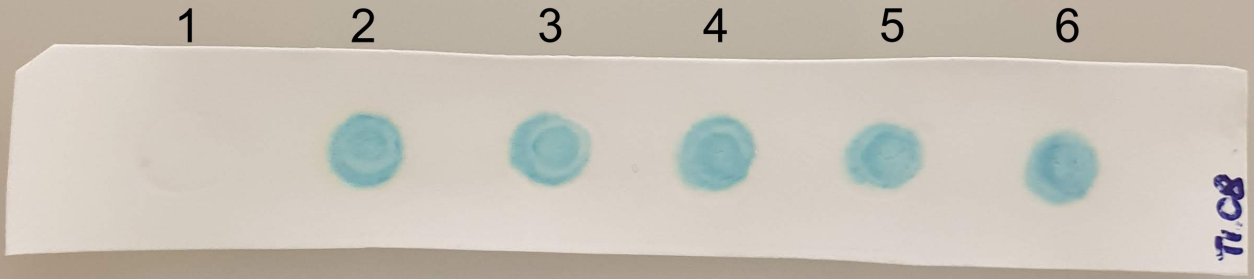

Figure 3d, 1.membrane-TKY2.T1.08. (1) 0 nM (2) 50 nM (3) 100 nM (4) 250 nM (5) 500 nM (6) 1000 nM. HRP-treated membrane was incubated with TMB substrate and image was captured by LCD Camera

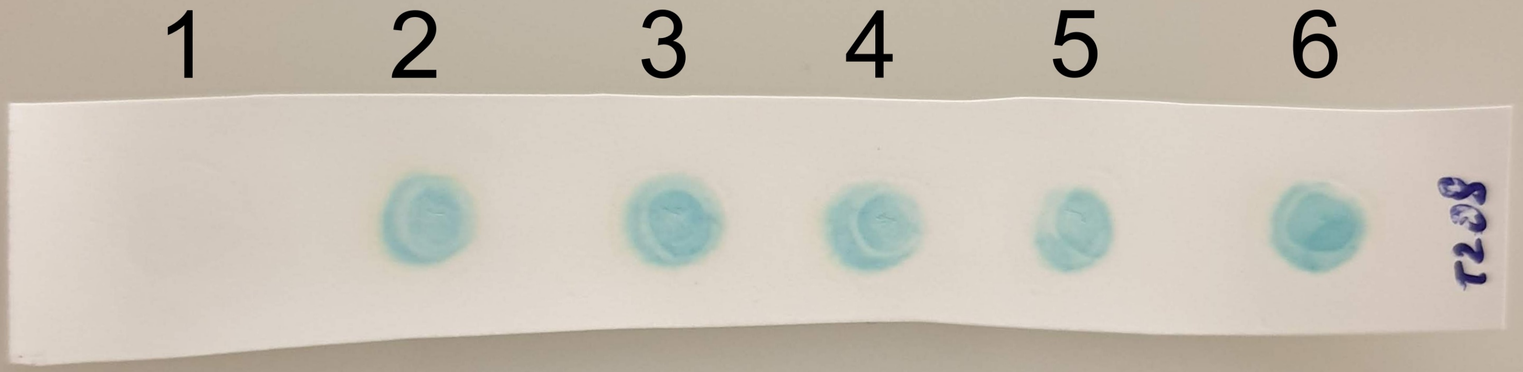

Figure 3d, 2. membrane-TKY.T2.08. (1) 0 nM (2) 50 nM (3) 100 nM (4) 250 nM (5) 500 nM (6) 1000 nM. HRP-treated membrane was incubated with TMB substrate and image was captured by LCD Camera

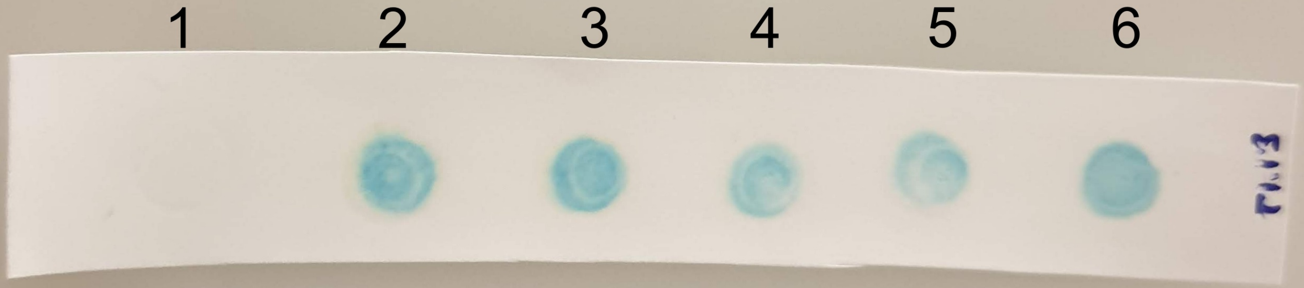

Figure 3d, 3.membrane-TKY2.T1.13. (1) 0 nM (2) 50 nM (3) 100 nM (4) 250 nM (5) 500 nM (6) 1000 nM. HRP-treated membrane was incubated with TMB substrate and image was captured by LCD Camera

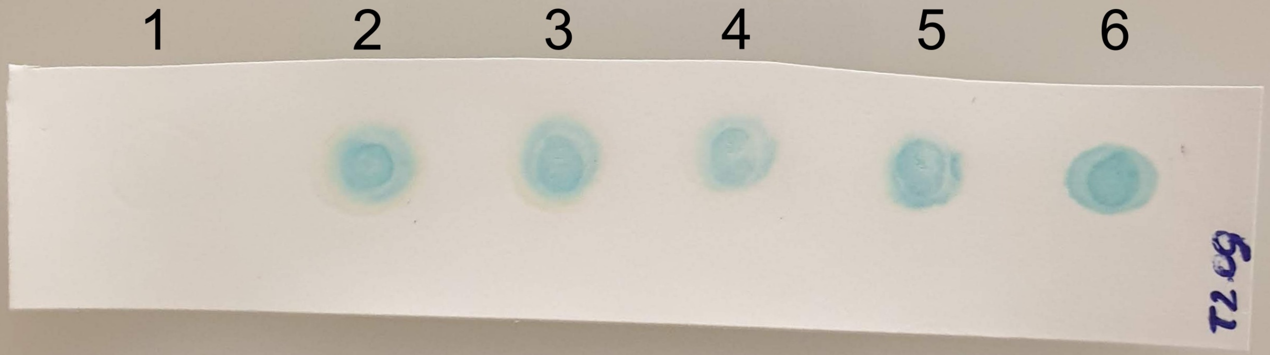

Figure 3d, 4.membrane-TKY.T2.09. (1) 0 nM (2) 50 nM (3) 100 nM (4) 250 nM (5) 500 nM (6) 1000 nM. HRP-treated membrane was incubated with TMB substrate and image was captured by LCD Camera

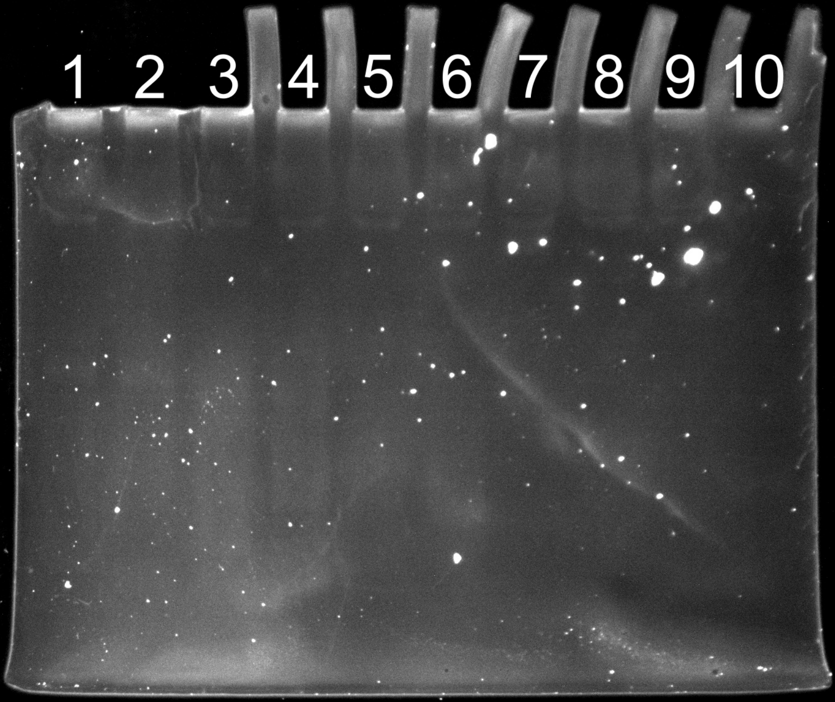

Figure 3e, TKY2.T1.01. (1) 0 h (2) 1 h (3) 3 h (4) 6 h (5) 24 h (6) 48 h (7) 72 h (8) 96 h (9) 120 h (10) + control. Polyacrylamide gel image was captured under UV light by UV Imaging System

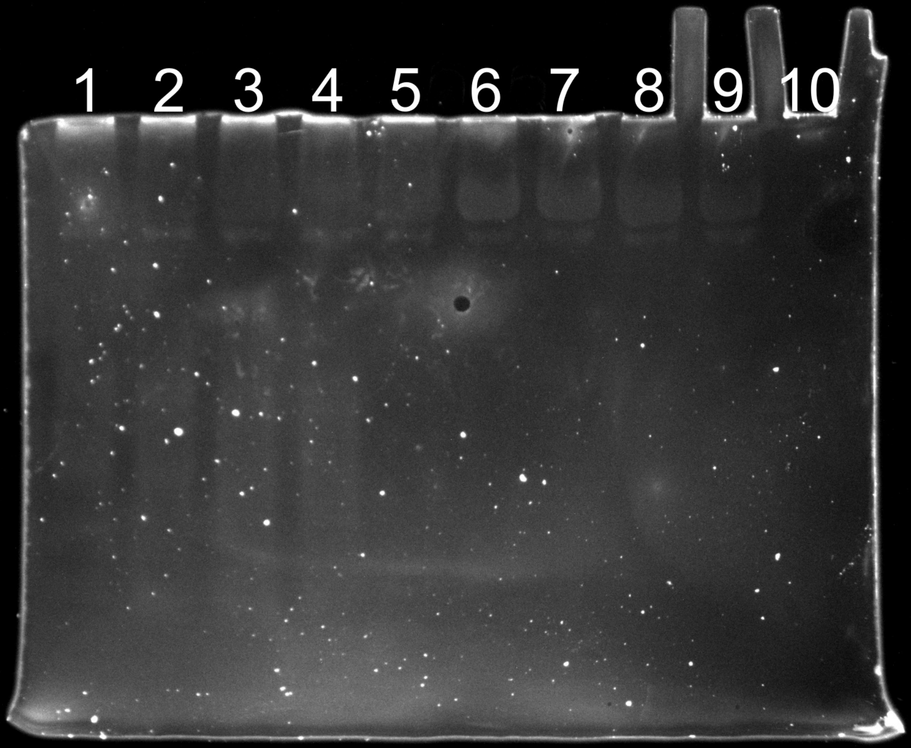

Figure 3e, TKY2.T1.02. (1) 0 h (2) 1 h (3) 3 h (4) 6 h (5) 24 h (6) 48 h (7) 72 h (8) 96 h (9) 120 h (10) + control. Polyacrylamide gel image was captured under UV light by UV Imaging System

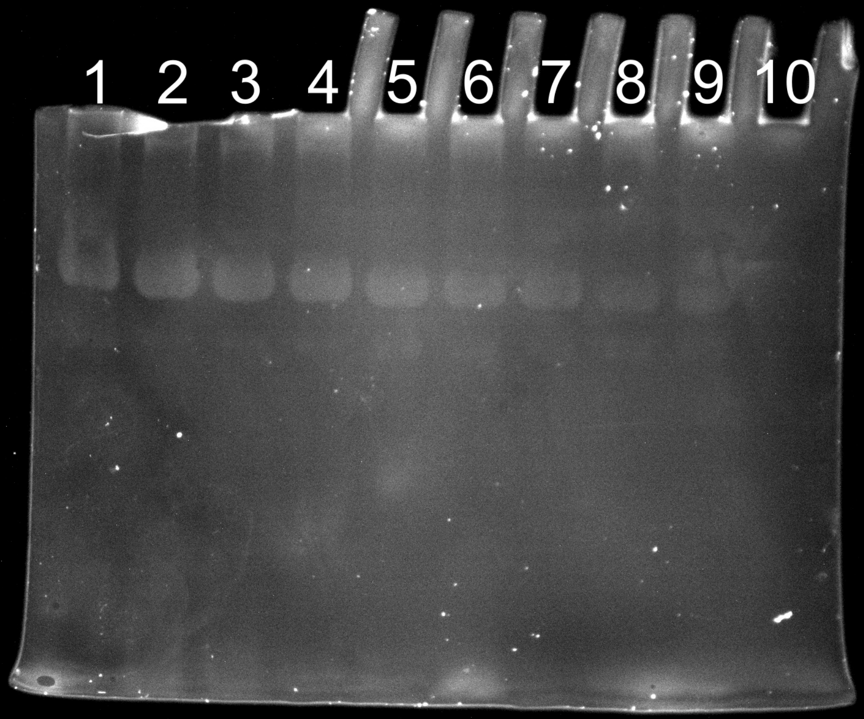

Figure 3e, TKY2.T1.03. (1) 0 h (2) 1 h (3) 3 h (4) 6 h (5) 24 h (6) 48 h (7) 72 h (8) 96 h (9) 120 h (10) + control. Polyacrylamide gel image was captured under UV light by UV Imaging System

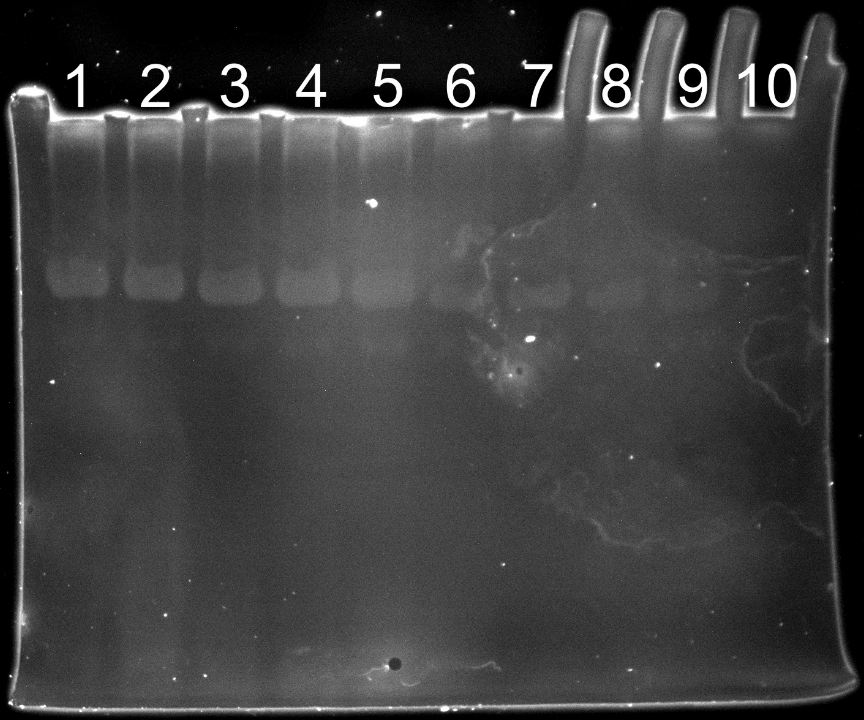

Figure 3e, TKY2.T1.04. (1) 0 h (2) 1 h (3) 3 h (4) 6 h (5) 24 h (6) 48 h (7) 72 h (8) 96 h (9) 120 h (10) + control. Polyacrylamide gel image was captured under UV light by UV Imaging System

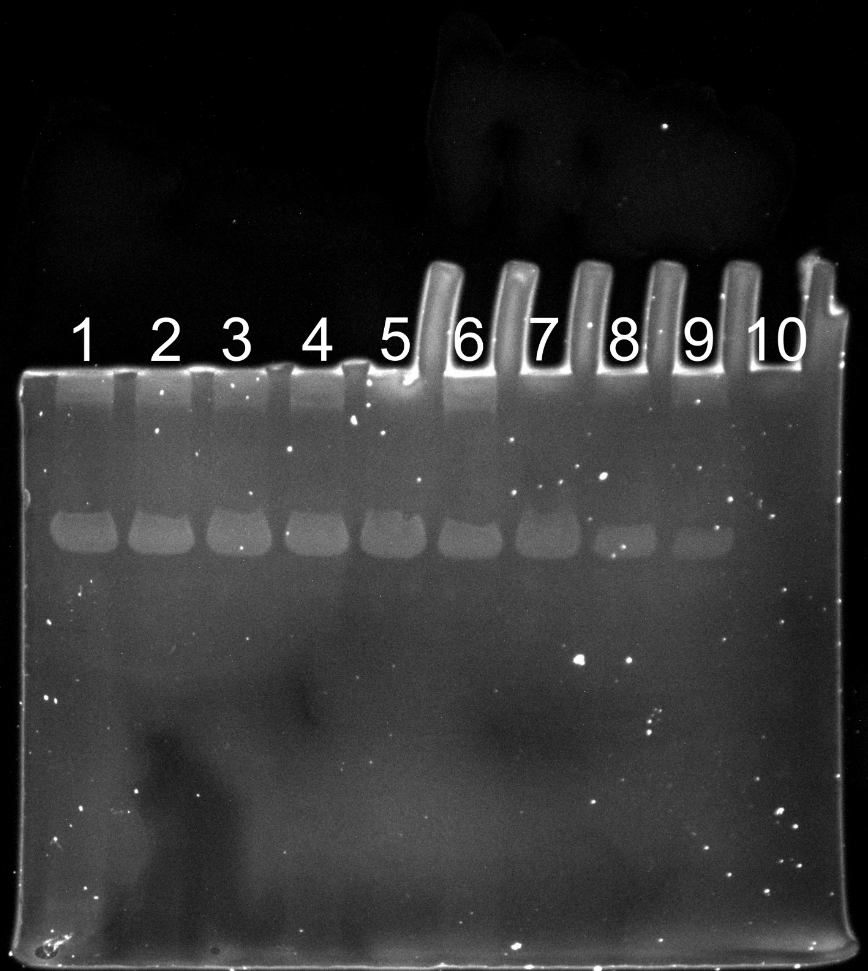

Figure 3e, TKY2.T1.05. (1) 0 h (2) 1 h (3) 3 h (4) 6 h (5) 24 h (6) 48 h (7) 72 h (8) 96 h (9) 120 h (10) + control. Polyacrylamide gel image was captured under UV light by UV Imaging System

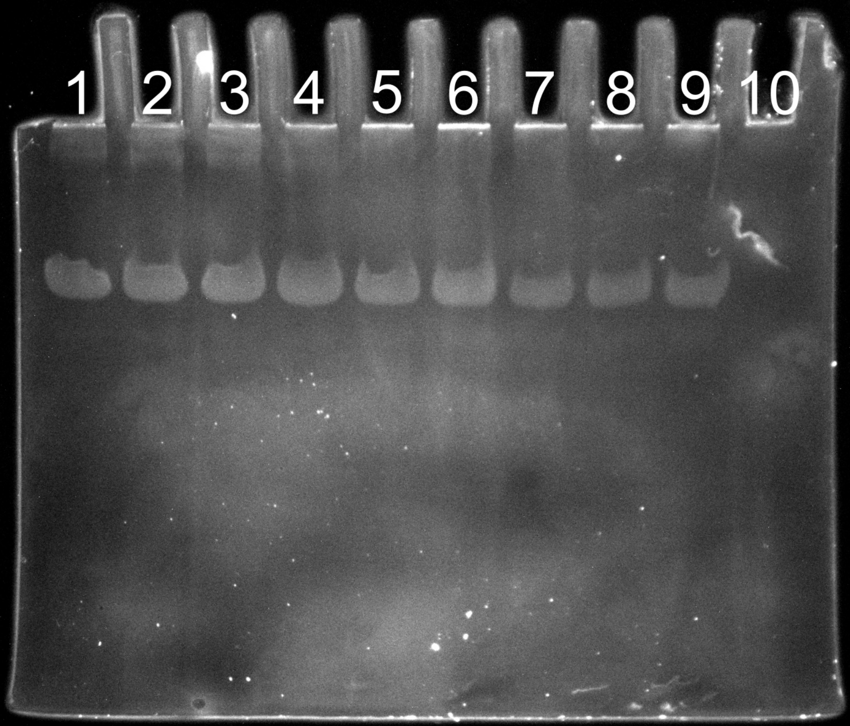

Figure 3e, TKY2.T1.06. (1) 0 h (2) 1 h (3) 3 h (4) 6 h (5) 24 h (6) 48 h (7) 72 h (8) 96 h (9) 120 h (10) + control. Polyacrylamide gel image was captured under UV light by UV Imaging System

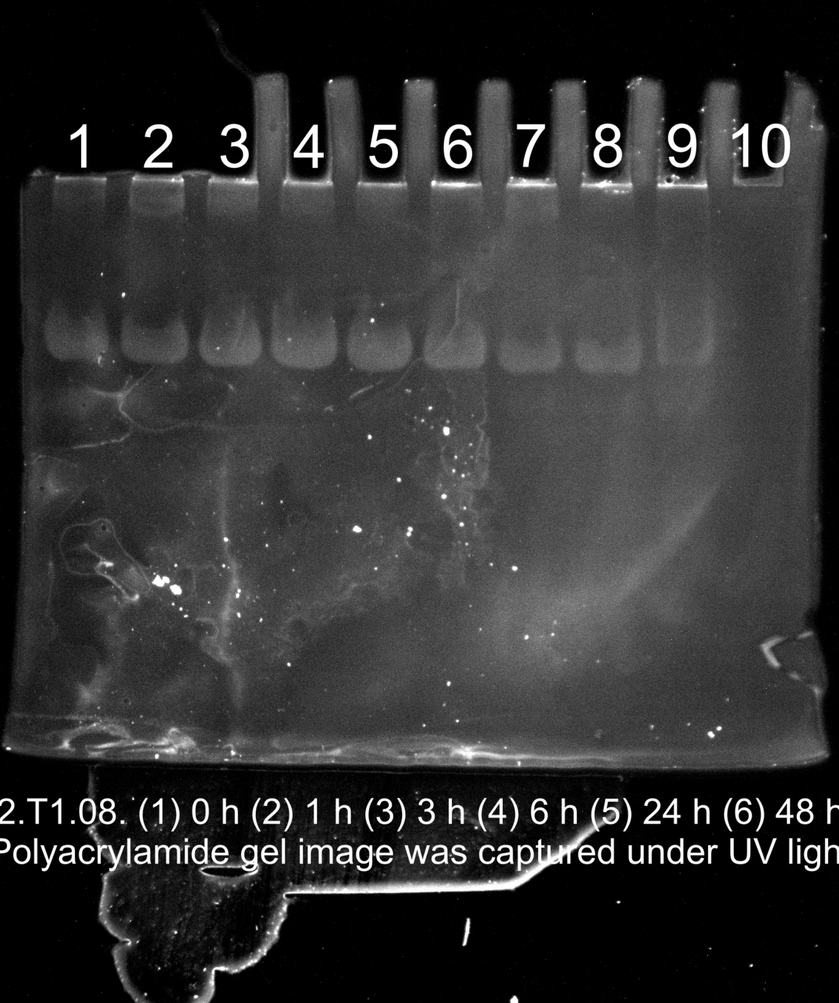

Figure 3e, TKY2.T1.08. (1) 0 h (2) 1 h (3) 3 h (4) 6 h (5) 24 h (6) 48 h (7) 72 h (8) 96 h (9) 120 h (10) + control. Polyacrylamide gel image was captured under UV light by UV Imaging System

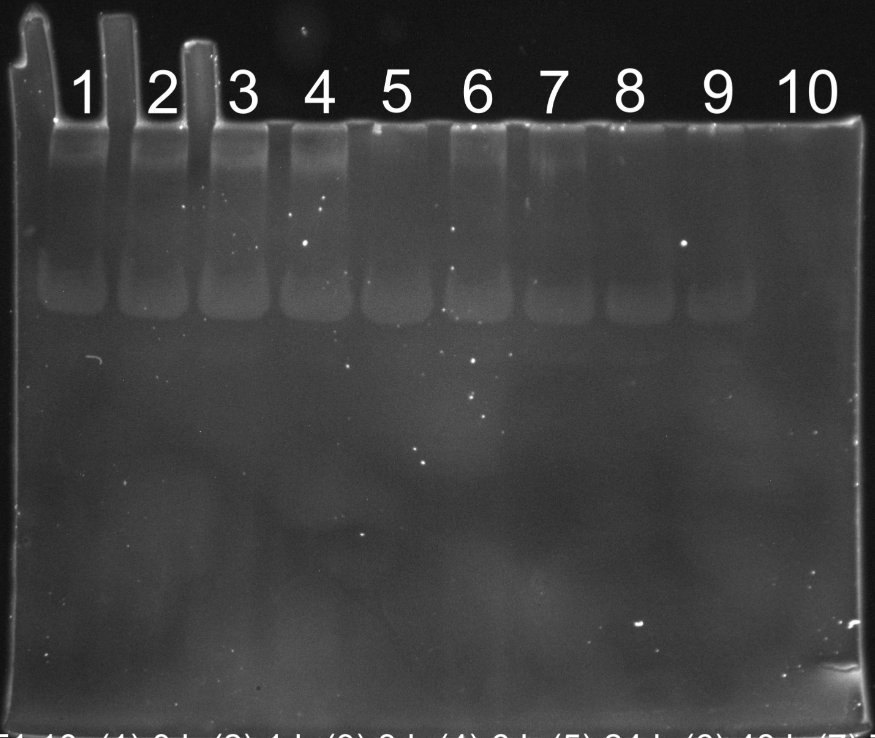

Figure 3e, TKY2.T1.10. (1) 0 h (2) 1 h (3) 3 h (4) 6 h (5) 24 h (6) 48 h (7) 72 h (8) 96 h (9) 120 h (10) + control. Polyacrylamide gel image was captured under UV light by UV Imaging System

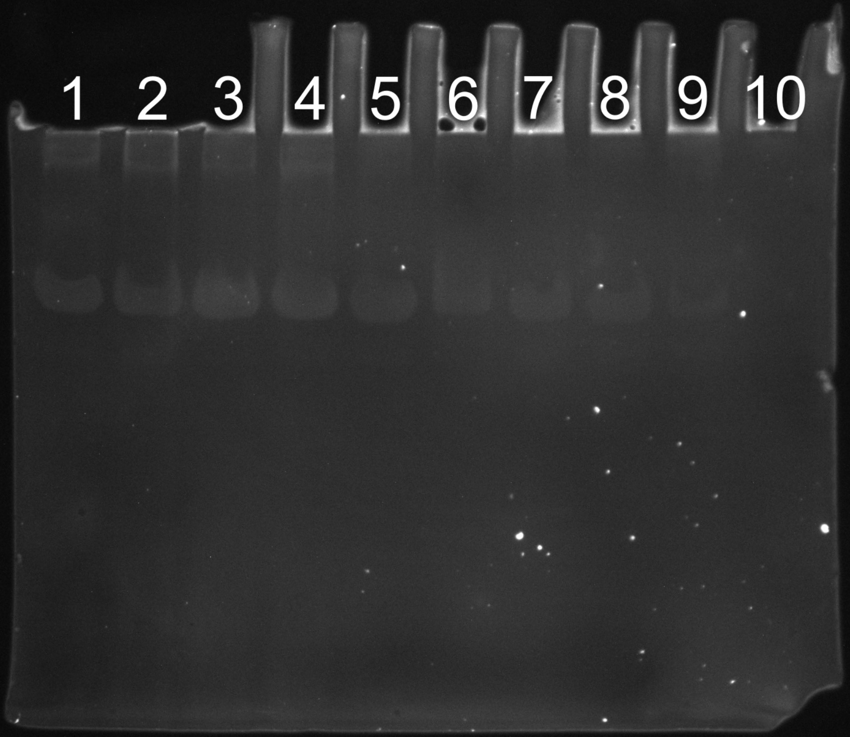

Figure 3e, TKY2.T1.11. (1) 0 h (2) 1 h (3) 3 h (4) 6 h (5) 24 h (6) 48 h (7) 72 h (8) 96 h (9) 120 h (10) + control. Polyacrylamide gel image was captured under UV light by UV Imaging System

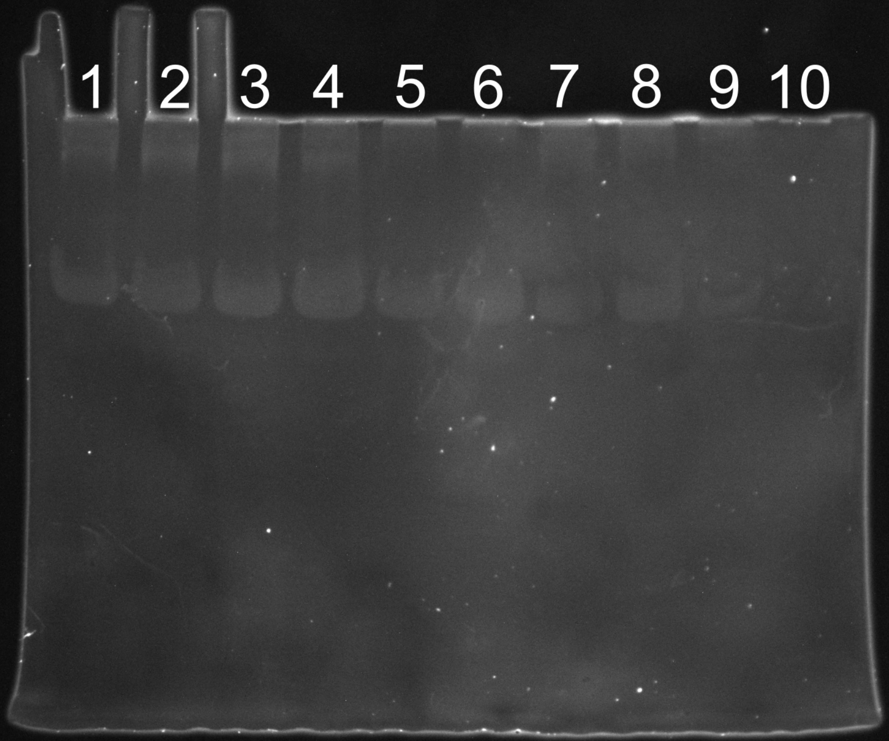

Figure 3e, TKY2.T1.12. (1) 0 h (2) 1 h (3) 3 h (4) 6 h (5) 24 h (6) 48 h (7) 72 h (8) 96 h (9) 120 h (10) + control. Polyacrylamide gel image was captured under UV light by UV Imaging System

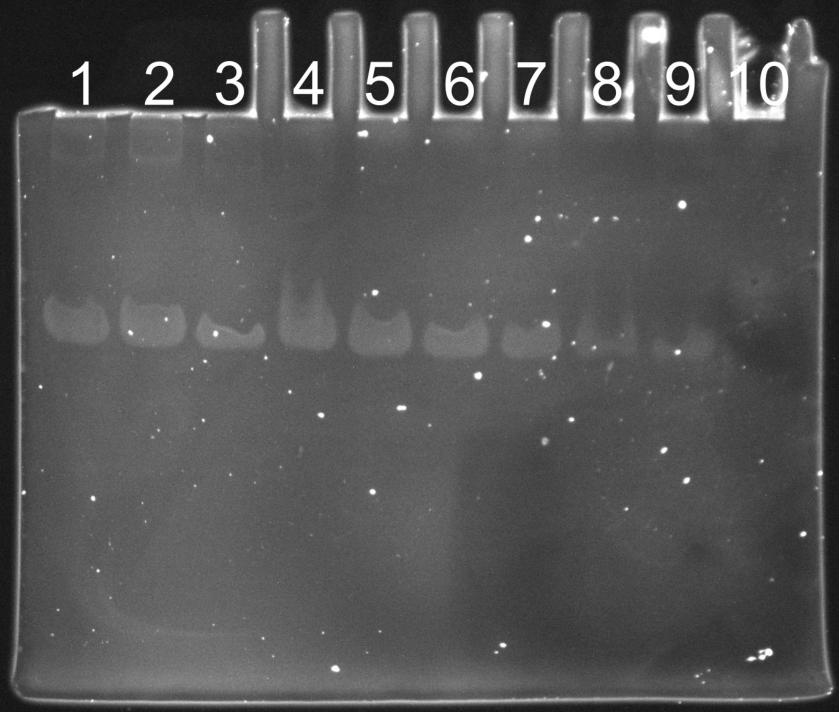

Figure 3e, TKY2.T1.13. (1) 0 h (2) 1 h (3) 3 h (4) 6 h (5) 24 h (6) 48 h (7) 72 h (8) 96 h (9) 120 h (10) + control. Polyacrylamide gel image was captured under UV light by UV Imaging System

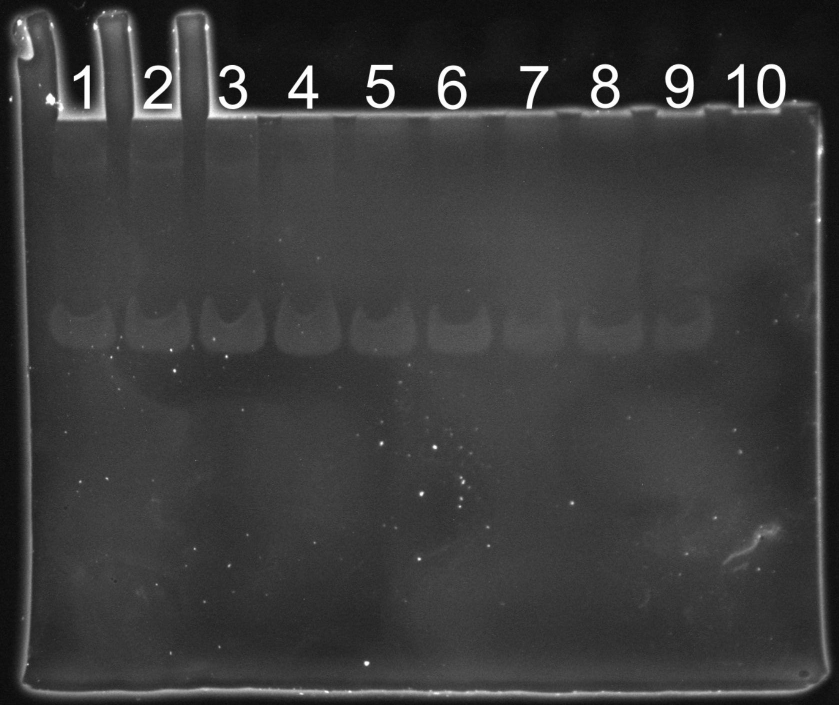

Figure 3e, TKY2.T1.15. (1) 0 h (2) 1 h (3) 3 h (4) 6 h (5) 24 h (6) 48 h (7) 72 h (8) 96 h (9) 120 h (10) + control. Polyacrylamide gel image was captured under UV light by UV Imaging System

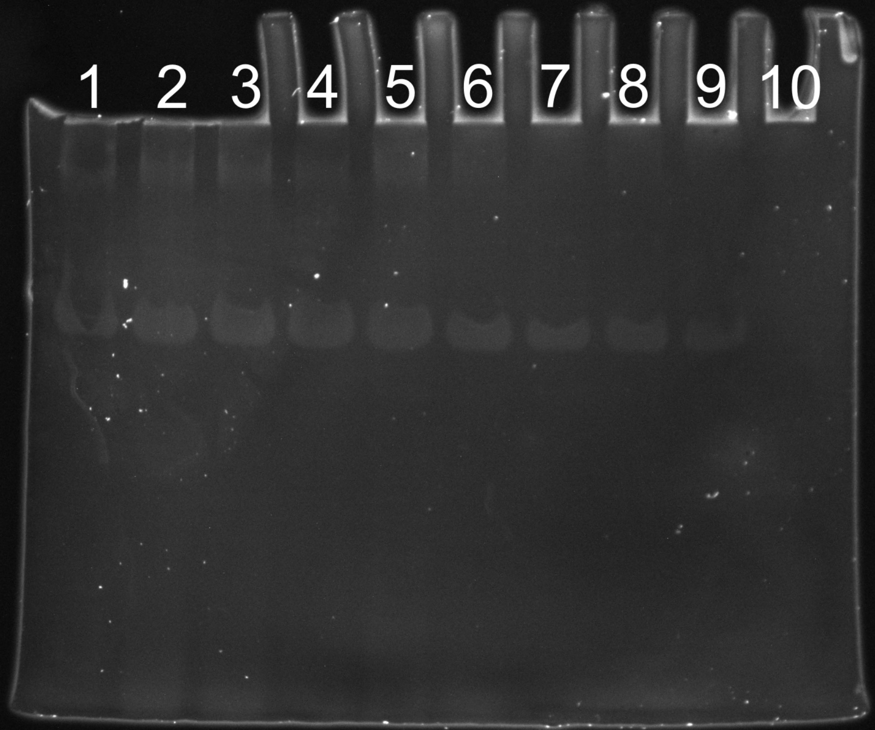

Figure 3e, TKY2.T1.16. (1) 0 h (2) 1 h (3) 3 h (4) 6 h (5) 24 h (6) 48 h (7) 72 h (8) 96 h (9) 120 h (10) + control. Polyacrylamide gel image was captured under UV light by UV Imaging System

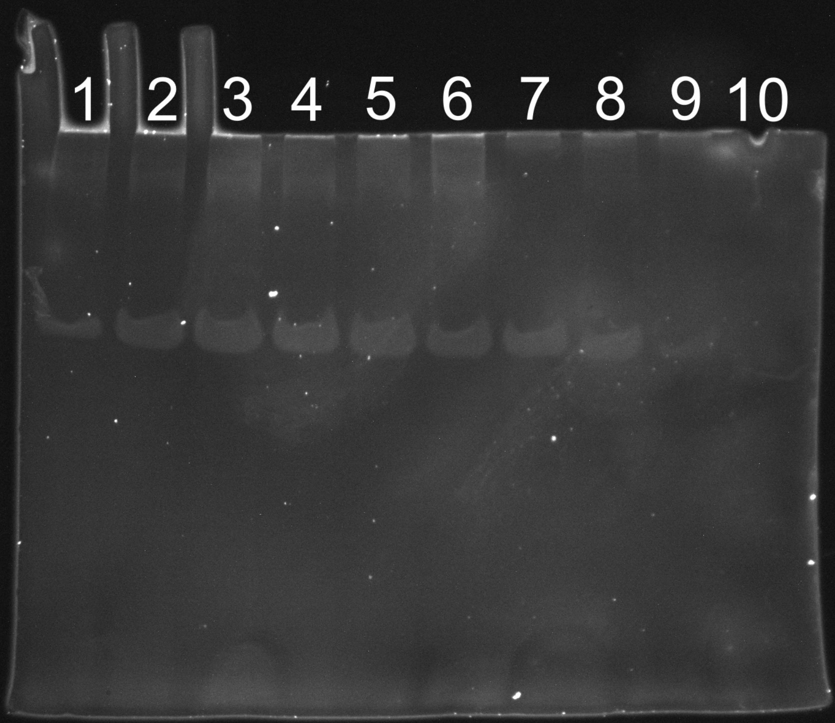

Figure 3e, TKY2.T1.17. (1) 0 h (2) 1 h (3) 3 h (4) 6 h (5) 24 h (6) 48 h (7) 72 h (8) 96 h (9) 120 h (10) + control. Polyacrylamide gel image was captured under UV light by UV Imaging System

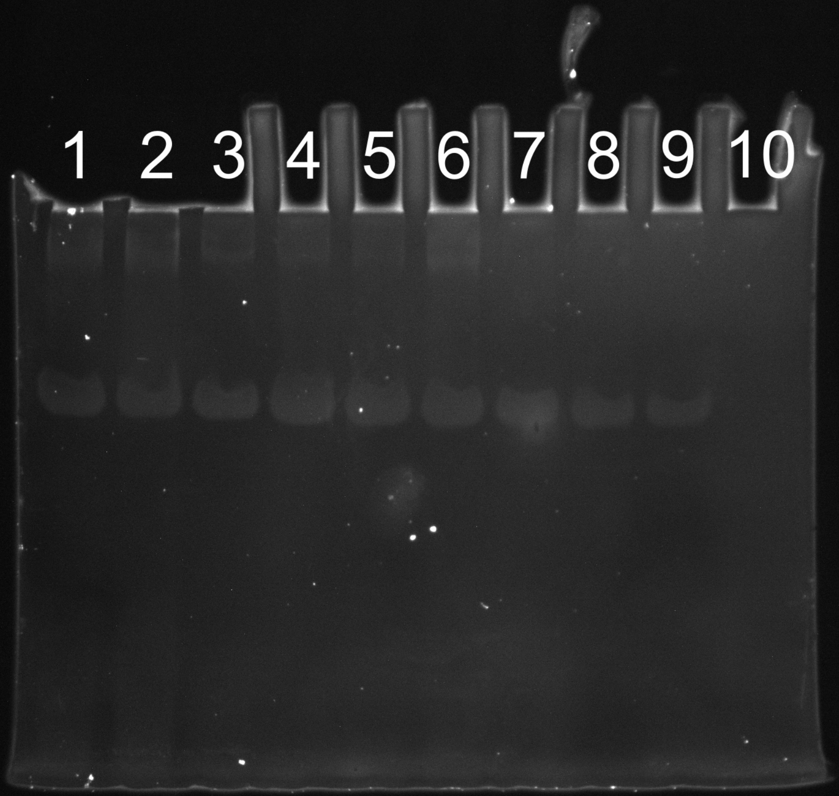

Figure 3e, TKY.T1.01. (1) 0 h (2) 1 h (3) 3 h (4) 6 h (5) 24 h (6) 48 h (7) 72 h (8) 96 h (9) 120 h (10) + control. Polyacrylamide gel image was captured under UV light by UV Imaging System

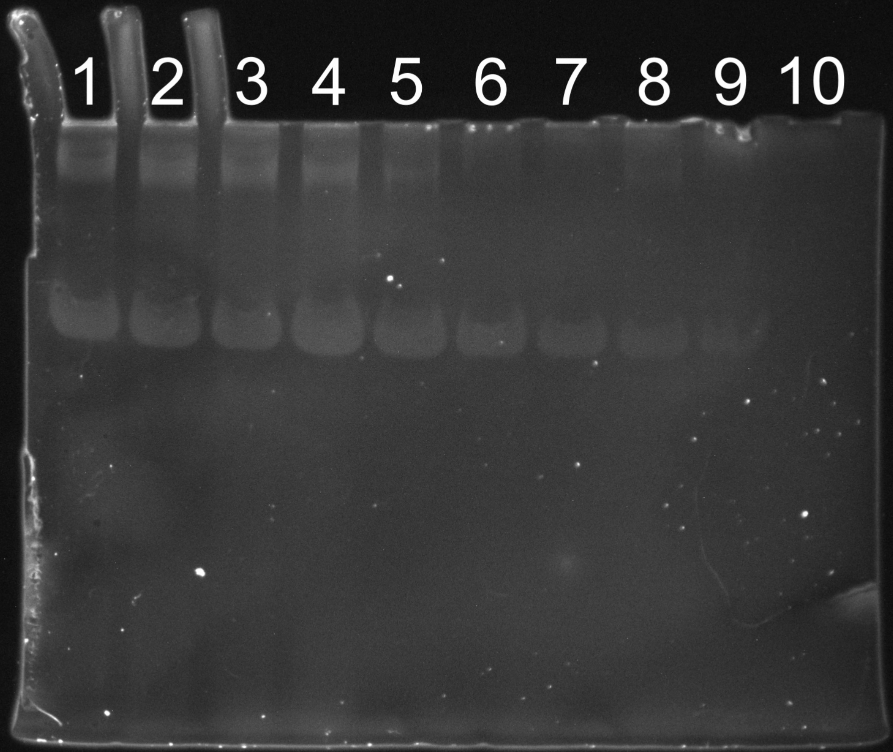

Figure 3e, TKY.T1.02. (1) 0 h (2) 1 h (3) 3 h (4) 6 h (5) 24 h (6) 48 h (7) 72 h (8) 96 h (9) 120 h (10) + control. Polyacrylamide gel image was captured under UV light by UV Imaging System

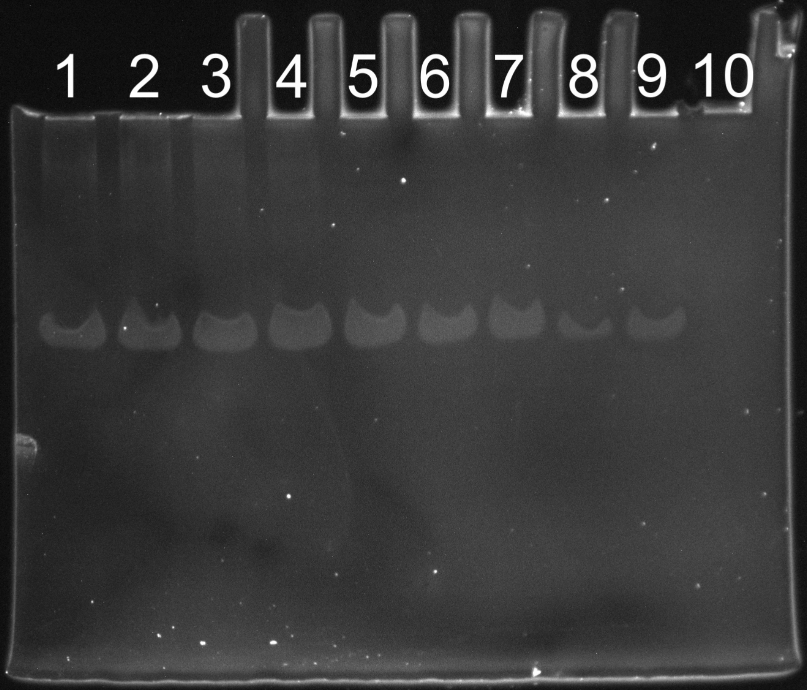

Figure 3e, TKY.T1.03. (1) 0 h (2) 1 h (3) 3 h (4) 6 h (5) 24 h (6) 48 h (7) 72 h (8) 96 h (9) 120 h (10) + control. Polyacrylamide gel image was captured under UV light by UV Imaging System

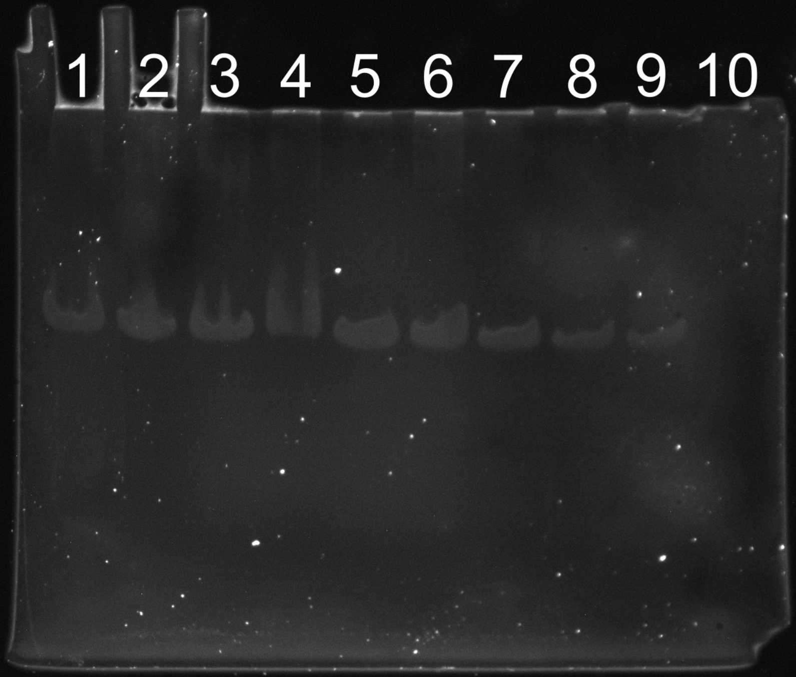

Figure 3e, TKY.T1.04 (1) 0 h (2) 1 h (3) 3 h (4) 6 h (5) 24 h (6) 48 h (7) 72 h (8) 96 h (9) 120 h (10) + control. Polyacrylamide gel image was captured under UV light by UV Imaging System

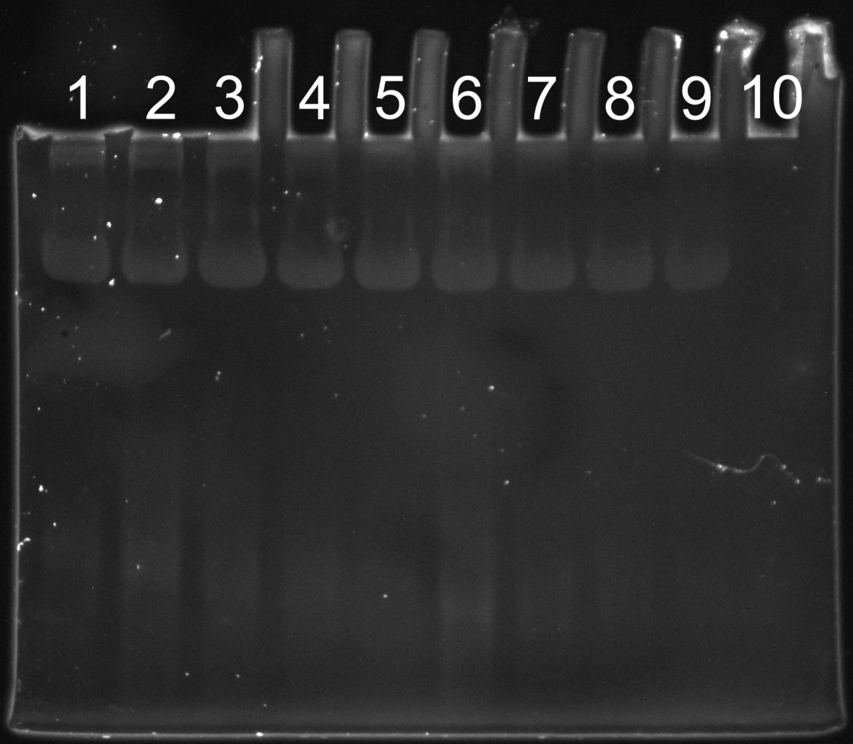

Figure 3e, TKY.T1.05. (1) 0 h (2) 1 h (3) 3 h (4) 6 h (5) 24 h (6) 48 h (7) 72 h (8) 96 h (9) 120 h (10) + control. Polyacrylamide gel image was captured under UV light by UV Imaging System

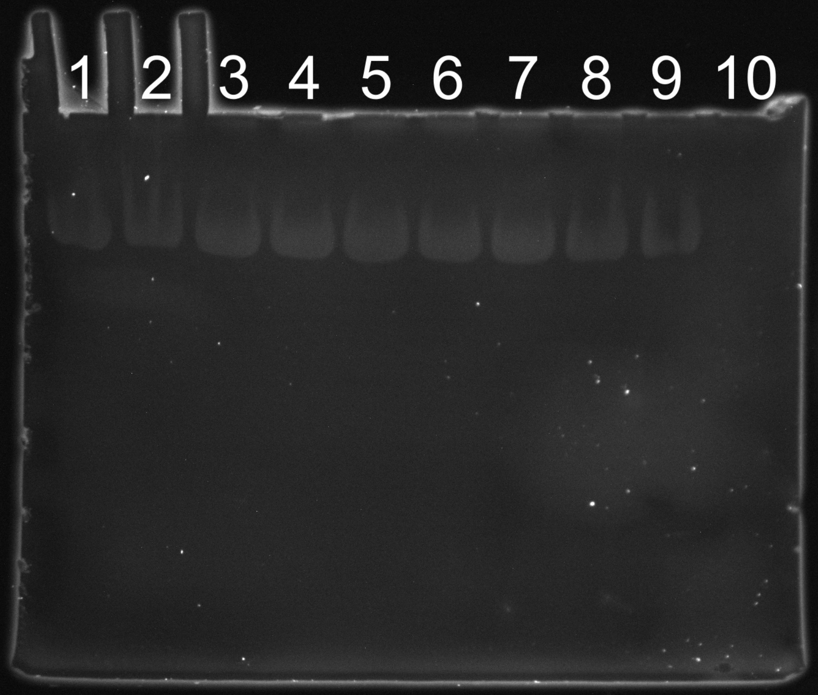

Figure 3e, TKY.T2.02. (1) 0 h (2) 1 h (3) 3 h (4) 6 h (5) 24 h (6) 48 h (7) 72 h (8) 96 h (9) 120 h (10) + control. Polyacrylamide gel image was captured under UV light by UV Imaging System

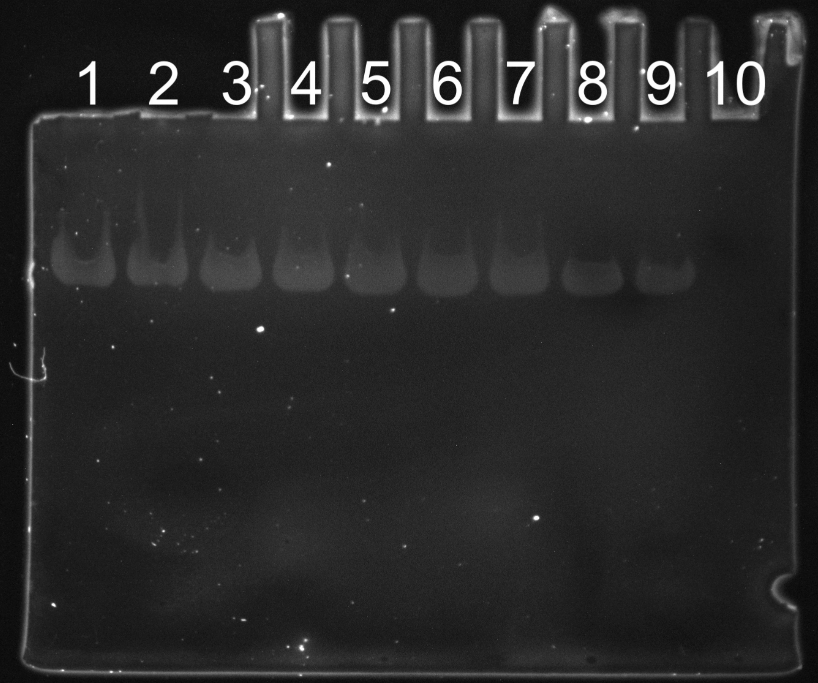

Figure 3e, TKY.T2.06. (1) 0 h (2) 1 h (3) 3 h (4) 6 h (5) 24 h (6) 48 h (7) 72 h (8) 96 h (9) 120 h (10) + control. Polyacrylamide gel image was captured under UV light by UV Imaging System

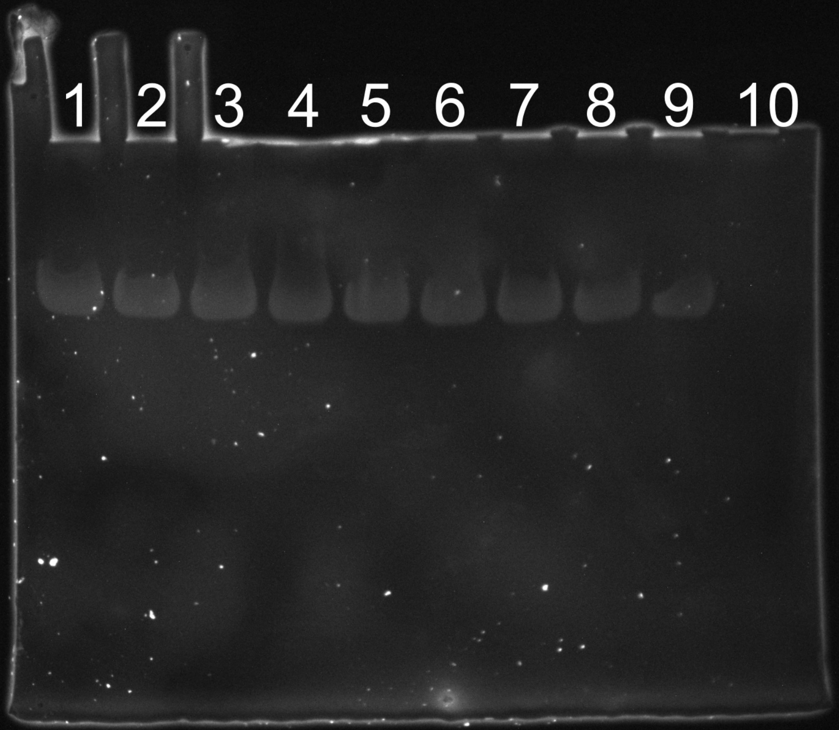

Figure 3e, TKY.T2.07. (1) 0 h (2) 1 h (3) 3 h (4) 6 h (5) 24 h (6) 48 h (7) 72 h (8) 96 h (9) 120 h (10) + control. Polyacrylamide gel image was captured under UV light by UV Imaging System

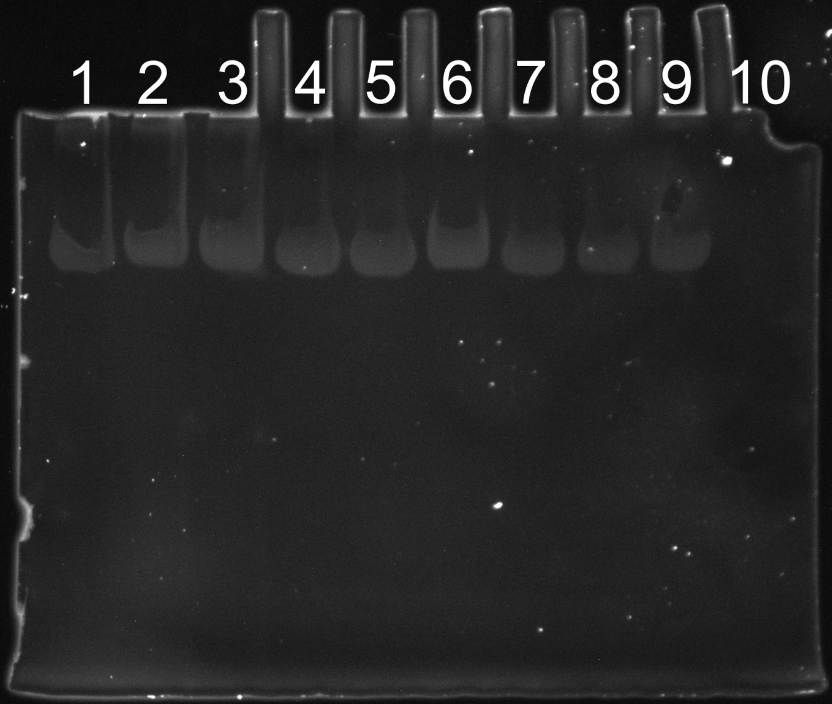

Figure 3e, TKY.T2.08. (1) 0 h (2) 1 h (3) 3 h (4) 6 h (5) 24 h (6) 48 h (7) 72 h (8) 96 h (9) 120 h (10) + control. Polyacrylamide gel image was captured under UV light by UV Imaging System

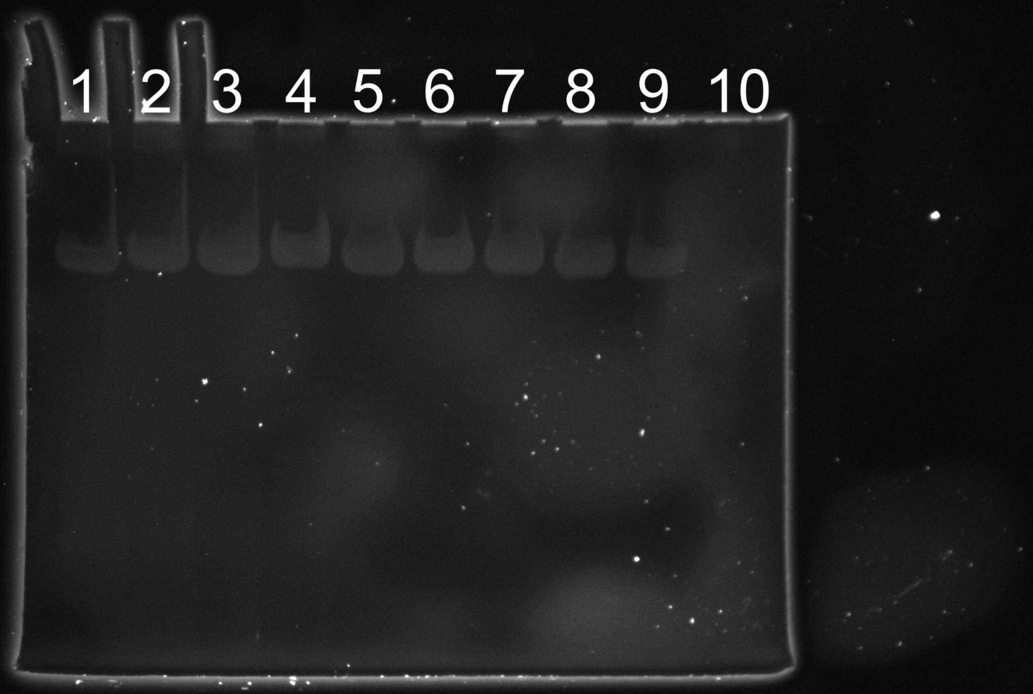

Figure 3e, TKY.T2.09. (1) 0 h (2) 1 h (3) 3 h (4) 6 h (5) 24 h (6) 48 h (7) 72 h (8) 96 h (9) 120 h (10) + control. Polyacrylamide gel image was captured under UV light by UV Imaging System

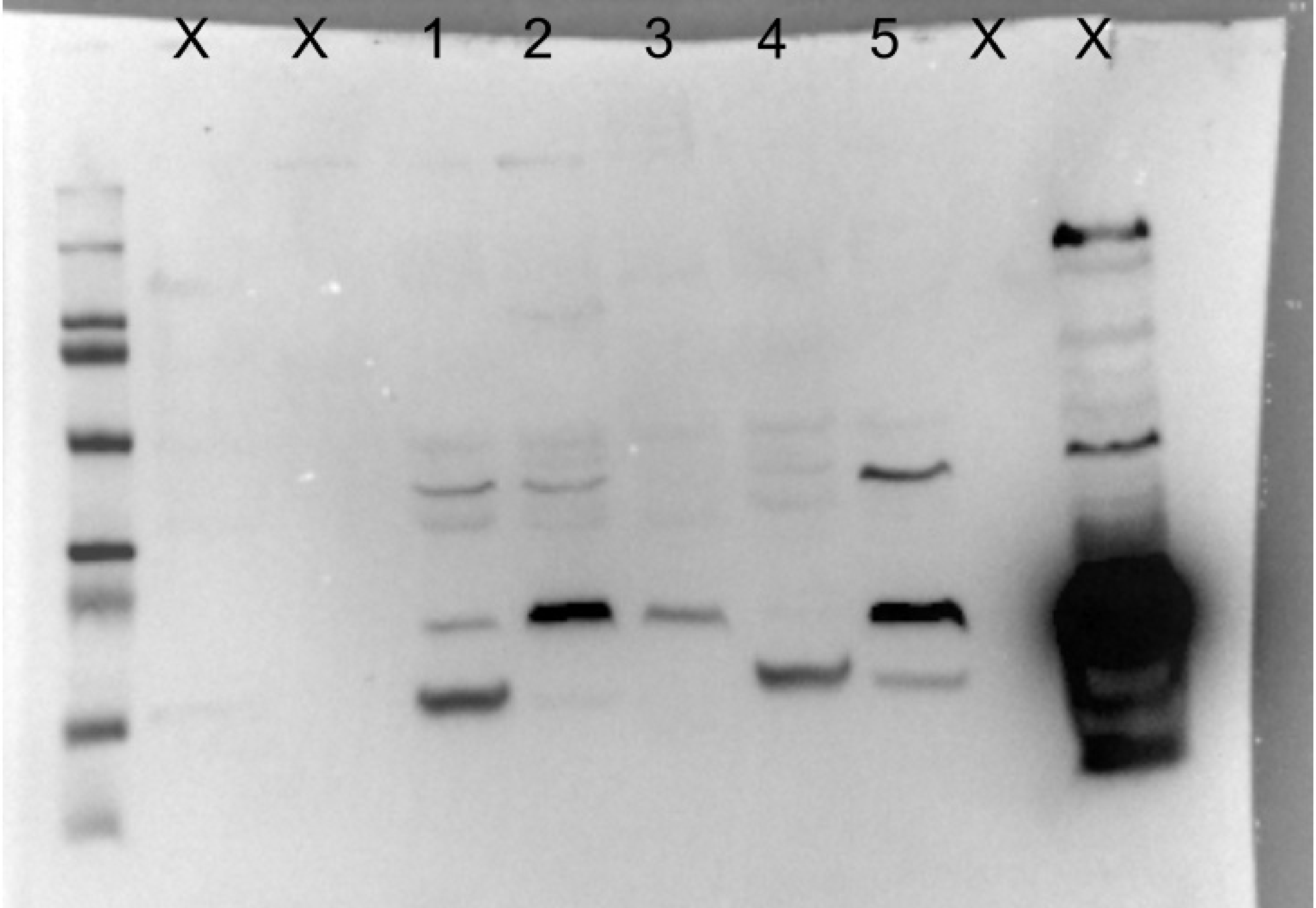

Figure 4b, GHRH (1) MIA PaCa-2 (2) HT29 (3) LNCaP (4) PNT1a (5) PC3. The membrane was captured by ChemiDoc MP Imaging System after Chemiluminescence staining

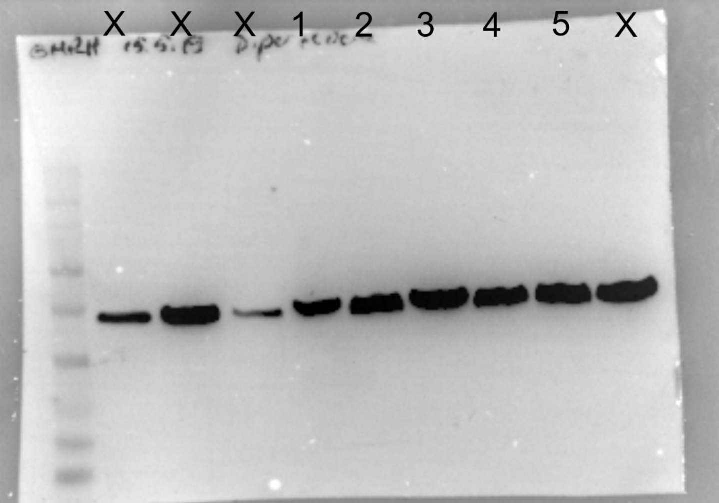

Figure 4b, ß-actin. (1) MIA PaCa-2 (2) HT29 (3) LNCaP (4) PNT1a (5) PC3. The membrane was captured by ChemiDoc MP Imaging System after Chemiluminescence staining
