# Supplementary material for: Synthesis and characterization of novel ssDNA X-aptamers targeting Growth Hormone Releasing Hormone (GHRH)
Source: PLoS One. 2022 Jan 21;17(1):e0260144. doi: 10.1371/journal.pone.0260144 (PMC8782341; doi:10.1371/journal.pone.0260144)
Supplement: Attachment — Submitted filename: Responsetoreviewers.docx [file pone.0260144.s002.docx]

Review Comments to the Author

Please use the space provided to explain your answers to the questions above. You may also include additional comments for the author, including concerns about dual publication, research ethics, or publication ethics. (Please upload your review as an attachment if it exceeds 20,000 characters)

Reviewer #1: This work focuses on the synthesis and purification of X-aptamers against Growth Hormone Releasing Hormone (GHRH), which was expressed in HEK293 and bacteria cells. GHRH is a hypothalamic neuropeptide that stimulates the production of growth factor GH, which in turn stimulates cell proliferation and division in different types of cancer.
24 X-aptamers were synthesized and 3 of them (TKY1.T1.13, TKY.T2.08, TKY.T2.09) had the best stability in serum and the highest binding potential by GHRH with low levels of Kd.

The work carried out is original, it has great relevance. It is well written and in my opinion meets the requirements to be accepted. However, some minor details need to be corrected.

***Comment 1:*** the quality of the figures must be corrected.

***Response to comment :*** All the figures quality is corrected

***Comment 2 :*** figure 2E does not match between title and file. There is no mention of this figure in the text. It also seems unnecessary to me since it is redundant with the 3D figüre

***Response to comment :*** According to reviewer’s comment about the figure 2e that was not matched with the title the mentioned figure was discarded from the revised version of the manuscript.

***Comment 3:*** Figure 5a-5h should be considered as supplementary material since the main purpose of the work is not to determine the ability of aptamers to bind to the cell lines studied
***Response to comment :*** According to other reviewer’s comment about figure 5a-5h, these figures couldn’t substituted to supplementary material. We performed GHRH siRNA transfection assays for this figure in order to demonstrate the effective binding of aptamers with GHRH ligand.

***Comment 4:*** Additional experiments are necessary to determine the ability of the obtained aptamers to block GHRH signaling. or at least make it clear that these experiments were not done (Page 19, lines 304-305 it is mentioned that aptamers are used to block GHRH).
***Response to comment :*** According to reviewer’s comment, the cAMP assay and coimmunofluorescence assays were performed. As the major downstream target of GHRH signaling were cAMP levels and target gene expressions (GH, GHRHR), we selected these experiments. Due to cAMP assay, significant decrease in cAMP levels were observed following selected aptamer treatment in HT29 and MIA Paca2 cells. Moreover, both GH and GHRHR expression profile after aptamers treatment was determined in MIA Paca-2 cells. All the results were inserted in the revised manuscript.

***Comment 5:*** make clear the limitations of the study

***Response to comment :*** In our previous version we tried to make clear the limitations of study by giving as “Finally, further studies may need to figure out the binding site of TKY2.T1.08, TKY2.T1.13, and TKY.T2.08 aptamers on GHRH epitope regions and determine the docking site for GHRHR and GHRH during GHRH-aptamer complex formation. Besides, *in silico* analysis, *in vitro* analysis might highlight the inhibitive effect of TKY2.T1.08, TKY2.T1.13, and TKY.T2.08, TKY.T2.09 X-aptamers on GHRH signaling.” And also in conclusion part we added “However, new studies might be performed in order to figure out the potential inhibitive effect of these x-aptamers on GHRH signaling and determination of docking sites for x-aptamers on GHRH ligands. In addition, further in vitro, in vivo and in silico analysis may need to highlight the impact of these three-novel x-aptamers efficiency on GHRH signaling.”

Reviewer #2: The authors report the development of aptamers selectively binding to the GHRH peptide and show binding efficiency by dot blot and SPR assays as well as by immunofluorescence assays with cells.
Issues to be addressed:

***Comment 1.*** Overall: Image quality is low

***Response to comment :*** Low quality images were corrected.

***Comment 2.*** Where should be an explanation for X-aptamers, and not just a reference. Can X aptamer sequences by modeled by using the mFOLD software?

***Response to comment :*** According to reviewer’s comment, instead of giving explanation for X-aptamer, we gave also reference in the revised version of the manuscript. Due to first reviewer the secondary structure of aptamers is not linked with the title so we discarded the 3D structure figures.

***Comment 3.*** How many SELEX cycles were performed? Why so many PCR cycles were performed, which indeed lead to the accumulation of PCR artefacts and not of selected aptamers. Was there any problem with the efficiency of the PCR reaction? The sequenced aptamers have a long 5` terminal T extension, I would guess that this an artefact. I suggest to align the aptamer consensus sequences in Figure 2C and make sure, which sequences belong to constant and variable sequences of the aptamers. Figure 2D should show, where in the loop region the consensus sequences can be found, and most importantly, the sequences should be readable. Figure 2E shows the stability of DNA aptamers along time. As this figure stands, it is not worth anything. First DNA needs to be resolved in a high-resolution denaturing polyacrylamide gel (which could distinguish between different sizes of aptamers); second, I do not see any positive controls for this assay (i.e. nuclease, which should degrade the aptamers).
***Response to comment :*** Due to X-aptamer synthesis kit instructions we performed increasing number of PCR amplifications in order to increase the yield of aptamers that are specific to GHRH peptide. So we performed 14, 22, 25 cycle of binding the magnetic bead based library with GHRH peptide. As this is a patented technology for generation of X-aptamers by kit based selection so accumulation of PCR artifacts were get rid of by each step washing procedure as every PCR cycle product was used as a template library for each following SELEX step. (https://www.youtube.com/watch?v=CgfX-KmXdA0). After amplifications, AM Biotechnologies were generated NGS in order to determine the novel putative sequences instead the ones similar with the 5’ and 3’ library linker sequences. (<https://www.genomeweb.com/resources/new-product/am-biotechnologies-x-aptamer-selection-kit>). All the sequences aligned and T rich 5’ region with “CACGAC” repeats were named as 5’ constant sequence and “GTGGGGCCCATG” sequence repeat at the 3’ region were named as 3’ constant sequence. The sequences between these fixed sequences were named as “variable sequence” . For the figure 2d, according to valuable contribution, we changed the figure to more readable one in the revised version of the text. According to comment on figure 2e, we performed polyacrylamide gel electrophoresis for serum stability of each aptamer and changed all the figures with the new results. Moreover, in each serum stability gel results, we added positive control as DNase I digested aptamers.

***Comment 4.*** The following experiment has a problem: "Then binding affinity of X-aptamers to target was assessed in increasing aptamer doses. Fold of change was calculated according to the dot intensities and dissociation constant (Kd) was calculated by nonlinear regression". Such binding data contain specific and unspecific binding. A competitor in excess concentration needs to be used in order to correct for unspecific binding at all aptamer doses.

***Response to comment :*** According to valuable contribution, we performed GHRH sandwich ELISA assay and dot blot analysis in order to detect the specifity of x-aptamers in dose-dependent manner for selected aptamers. The performed experiment results were given in Figure 3d. As one of most effective competitor for aptamer is antibody, we preffer to perform GHRH ELISA assay. GHRH ELISA is a sandwich ELISA and wells were coated with GHRH antibody. 500 nM GHRH peptide is applied to wells and dose-dependent aptamers was used as they are 5’ biotinylated in order to determine the colorimetric analysis. And we performed dot-blot from supernatant of GHRH-aptamer complex in dose-dependent manner for selected x-aptamers. Thus, we demonstrated dose-dependent x-aptamer is a competitor for GHRH antibody and x-aptamers bind to target GHRH peptide with a high binding affinity.

***Comment 5.*** A two or three of change of binding is very little, assuming that the unselected aptamer library shows unspecific binding in the mM range. Binding affinities of aptamers should increase at least 1,000 fold, reflected by more than of a two or three fold amount of target bound aptamers.

***Response to comment :*** According to valuable contribution, we repeated dot-blot analysis for all aptamers. And then calculated the fold of change due to colorimetric analysis of each spot comparing with the negative control as only GHRH ligand was used. The repeated experiment results and fold of change in GHRH binding affinity due to dot-blot analysis was replaced in the revised version of the manuscript.

***Comment 6.*** Negative controls of immune reactions, including those with aptamers are missing.

***Response to comment :*** According to valuable contribuition, we applied GHRH siRNA as a negative control of immune reactions. Beside dose-dependent aptamer applications, we also apply aptamers in MIA Paca-2 cells with blocked GHRH signaling by siRNA. Due to experiments GHRH silencing as a negative control for immune reactions demonstrated that aptamers didn’t bind to MIA Paca-2 cells. The new experiment results were replaced in the figure 5b-d-f-h in the revised version of the figures.
