# Supplementary material for: Synthesis and characterization of novel ssDNA X-aptamers targeting Growth Hormone Releasing Hormone (GHRH)
Source: PLoS One. 2022 Jan 21;17(1):e0260144. doi: 10.1371/journal.pone.0260144 (PMC8782341; doi:10.1371/journal.pone.0260144)
Supplement: Attachment — Submitted filename: Rev.docx [file pone.0260144.s003.docx]

Introduction

The study presents the synthesis of 24 putative X-aptamers against GHRH peptides with high serum stability, target binding, and low Kd levels.

Overall:

English needs proofreading.

Figure legends should be more informative, otherwise, the reader has to look back and forth to find the information scattered in the manuscript.

Specific questions:

Comment#1 Introduction does not present clear information regarding the goal of making those aptamers. Do authors want to make GHRH antagonists? Authors want to make new therapeutic agents or diagnostic tools? It’s not clear and should be clarified in the Introduction section.

Comment#2 Why authors want to make aptamers against 1-44 and/or 1-29 peptides? Are there any advantages/differences of using each peptide target for selection? A better explanation in the Introduction regarding the reasons for testing both targets is desirable. The only thing that is mentioned is the fact that biological activity is observed in the 1-29 sequence. If so, why not only 1-29 protein was used as a target? Also, I could not find in the Discussion section, whether 1-44 targeting aptamers were also able to detect 1-29 protein and vice versa. I believe this should be addressed in the discussion.

Comment#3 X-aptamer technology was recently developed. The advantages and limitations of this SELEX procedure need to be further explored in the Introduction section.

Comment#4 Line 99, “Expression of bacterial His” needs to be further explained. Is it the control of the experiment? Also, plasmids pCMV3-SP-N-His-NCV and pCMV3-SP-His-ORF His-GHRH are not defined. Which proteins are coded by these plasmids? A detailed explanation of each construct is desirable.

Comment#5 Line 160. What is the composition of the selection buffer?

Comment#6 Line 173 “4 1.5 ml tubes”…needs improvement

Comment#7 Units are wrong throughout the whole manuscript ….for example μl needs to be replaced by μL, ml by mL, etc.

Comment# 8 Line 248-249. The sentence appears incomplete.

Comment#9 Line 253…” HEK293 cells were used…”

Comment#10 Methodology of aptamer selection is very confusing. Author state: “Our aim in this study is to synthesize and purify GHRH (1-44) protein in HEK293 cells, synthesize and select X-aptamers against GHRH peptide both 1-29 and 1-44, and demonstrate synthesized aptamers’ target binding activity as well as serum stability.”

If only purification of GHRH 1-44 protein was performed, how the selection was performed using both proteins (1-29 and 1-44) as targets? There were two different Selex procedures, each one using one protein as the target? That is not clear.

If authors claim that will select aptamers against both 1-29 and 1-44 peptides, the methodology should provide a detailed description of which peptide was used target for selection.

Also, seems strange that authors tried to express in E.coli since it’s widely known that bacteria lack a posttranscriptional modification mechanism. Line 252-254 says “Since the bacterial protein synthesis mechanism lacks post-translational modifications, HEK293 cells were used…” this information was already available before the bacterial expression was performed in this article, therefore, I wonder why bacterial expression was tested and included in the study…

Although using HEK293 for protein expression, it is not clear if 1-29 or 1-44 peptides were expressed.

Comment#11 Sequences provided in Figure 2 are aptamers obtained from which selection (1-29 or 1-44)? What is the difference between sequences named TKY2 and TKY?

Comment#12 Figure 1 shows expression and purification of NH2 (1-44) target or GHRH NH2 (1-29) target? Figure legends should be more informative, otherwise, the reader has to look back and forth to find the information scattered in the manuscript.

Comment#13. Bacterially expressed His-GHRH protein was expressed as inclusion bodies and denatured using Guanidine. No protocol for protein refolding was presented.
