# Supplementary material for: Synthesis and characterization of novel ssDNA X-aptamers targeting Growth Hormone Releasing Hormone (GHRH)
Source: PLoS One. 2022 Jan 21;17(1):e0260144. doi: 10.1371/journal.pone.0260144 (PMC8782341; doi:10.1371/journal.pone.0260144)
Supplement: Attachment — Submitted filename: Response_to_Reviews.docx [file pone.0260144.s004.docx]

Introduction

The study presents the synthesis of 24 putative X-aptamers against GHRH peptides with high serum stability, target binding, and low Kd levels.

Overall:

English needs proofreading.

- Manuscript English was corrected and some sentences were rewritten.

Figure legends should be more informative, otherwise, the reader has to look back and forth to find the information scattered in the manuscript.

- Figure legends were detailed and altered to more informative.

Specific questions:

**Comment#1 Introduction does not present clear information regarding the goal of making those aptamers. Do authors want to make GHRH antagonists? Authors want to make new therapeutic agents or diagnostic tools? It’s not clear and should be clarified in the Introduction section**.

***Reply to comment 1:*** *According to valuable comment, we explained in the introduction part that we want to make GHRH aptamer antagonists. And we added “In order to generate GHRH antagonists, we select aptamers due to their high binding affinity, low toxicity, non-immunogenic properties (11)” sentense after GHRH peptide antagonists in the revised text.*

**Comment#2 Why authors want to make aptamers against 1-44 and/or 1-29 peptides? Are there any advantages/differences of using each peptide target for selection? A better explanation in the Introduction regarding the reasons for testing both targets is desirable. The only thing that is mentioned is the fact that biological activity is observed in the 1-29 sequence. If so, why not only 1-29 protein was used as a target? Also, I could not find in the Discussion section, whether 1-44 targeting aptamers were also able to detect 1-29 protein and vice versa. I believe this should be addressed in the discussion.**

***Rept to comment 2:*** *We rewrote the introduction and discussion parts to explain why we use both peptides for aptamer synthesis. This is the first report demonstrating the synthesis of aptamers against GHRH so we want to generate SELEX against both 1-44 and 1-29. Like the same strategy with Macugen (anti-VEGF), we preferred to synthesize X-aptamers against GHRH full length protein which is 44 amino acid long. Moreover, this will give us to detect every epitopic part of GHRH variants when we synthesized aptamers. In addition, due to GHRH peptide antagonist that targets GHRH 1-29, we used 1-29 peptide as a target to demonstrate the impact of aptamers by comparing every epitopic region. We inserted “As there is not any GHRH antagonist aptamer, we preferred to synthesize aptamers against both GHRH 1-44 and 1-29. In order to synthesize X-aptamers that can capture every epitopic region, we selected full GHRH protein NH2 (1-44). Besides, as GHRH peptide antagonist select 1-29 region, we used GHRH NH2 (1-29) as a target in order to synthesize X-aptamers. In order to illustrate by comparing the binding affinity of GHRH antagonist X-aptamers, we aimed to synthesize, select X-aptamers against both GHRH NH2 (1-44) and NH2 (1-29) peptides.” Due to dot blot analysis, both GHRH NH2 (1-44) X-aptamers and GHRH NH2 (1-29) aptamers have similar binding affinity against GHRH peptide target. Thus, in discussion section we added “When we compared the binding affinity of GHRH NH2 (1-44) x-aptamers with GHRH NH2 (1-29) peptide, similar binding potential illustrated by GHRH NH2 (1-44) X-aptamers against GHRH (1-44) peptide in dot-blot analysis.”*

**Comment#3 X-aptamer technology was recently developed. The advantages and limitations of this SELEX procedure need to be further explored in the Introduction section.**

***Reply to comment 3:*** *The advantages and limitations of this SELEX procedure were given in the introduction part as “The most important advantage of X-aptamer technology is to synthesize up to 5 different target at the same time by one SELEX method. However, the limitation of X-aptamer technology is molecular size of target molecule, targets longer than 10 amino acids is assumed to be more preferred (14).”*

**Comment#4 Line 99, “Expression of bacterial His” needs to be further explained. Is it the control of the experiment? Also, plasmids pCMV3-SP-N-His-NCV and pCMV3-SP-His-ORF His-GHRH are not defined. Which proteins are coded by these plasmids? A detailed explanation of each construct is desirable.**

***Rept to comment 4:*** *The expression of bacterial His performed as a control in order to determine the His-tagged protein expression in HEK293 cells. His tagged protein synthesized by pCMV3-SP-N-His-NCV plasmid and His-tagged GHRH peptide expressed from pCMV3-SP-His-ORF His-GHRH plasmid. This explanation was given in the revised version of the manuscript.*

**Comment#5 Line 160. What is the composition of the selection buffer?**

***Reply to comment 5:*** *The composition of the selection buffer is given in the revised version of the manuscript.* *The content of selection buffer is 1X PBS pH:7.4, 1 mM MgCl2, 0.05% Tween20, 0.02% BSA.*

**Comment#6 Line 173 “4 1.5 ml tubes”…needs improvement**

***Reply to comment 6:*** “4 1.5 ml tubes” was changed to “four 1.5 mL tubes” in the revised manuscript.

**Comment#7 Units are wrong throughout the whole manuscript ….for example μl needs to be replaced by μL, ml by mL, etc.**

***Reply to comment 7:*** *We changed them all in the revised text.*

**Comment# 8 Line 248-249. The sentence appears incomplete.**

***Reply to comment 8:*** *According to valuable comment, the sentence* *appears to be incomplete was changed to “Purification of His-tagged GHRH peptide “ as a title for result section.*

**Comment#9 Line 253…” HEK293 cells were used…”**

***Reply to comment 9:*** *According to valuable comment,* *“HEK293 cells were used” changed the revised manuscript.*

**Comment#10 Methodology of aptamer selection is very confusing. Author state: “Our aim in this study is to synthesize and purify GHRH (1-44) protein in HEK293 cells, synthesize and select X-aptamers against GHRH peptide both 1-29 and 1-44, and demonstrate synthesized aptamers’ target binding activity as well as serum stability.”**

**If only purification of GHRH 1-44 protein was performed, how the selection was performed using both proteins (1-29 and 1-44) as targets? There were two different Selex procedures, each one using one protein as the target? That is not clear. If authors claim that will select aptamers against both 1-29 and 1-44 peptides, the methodology should provide a detailed description of which peptide was used target for selection.**

**Also, seems strange that authors tried to express in E.coli since it’s widely known that bacteria lack a posttranscriptional modification mechanism. Line 252-254 says “Since the bacterial protein synthesis mechanism lacks post-translational modifications, HEK293 cells were used…” this information was already available before the bacterial expression was performed in this article, therefore, I wonder why bacterial expression was tested and included in the study…**

**Although using HEK293 for protein expression, it is not clear if 1-29 or 1-44 peptides were expressed.**

***Reply to comment 10:*** *We realized that we didn’t write in the material method section that we used bacterial and eukaryotic GHRH NH2 (1-44) peptide and also GHRH NH2 (1-29) peptide as a target for X-aptamer SELEX. We performed two SELEX; first SELEX; bacterial/eukaryotic SELEX (Figure 2a) and second SELEX: GHRH 1-29 (figure 2b). As X-aptamer synthesing kit targets up to 5 targets, we used purified bacterial and eukaryotic GHRH 1-44 peptide for the first SELEX and also GHRH 1-29 peptide for the second SELEX. According to manufacturers instructions, target must be magnetically labelled with either his or streptavidin magnetic beads. Thus, we have to use His-tagged GHRH protein for his-magnetic beads and also biotinylated GHRH for streptavidin magnetic bead. Thus, in order to compare the binding affinity of X-aptamers against each target we prefer to use GHRH 1-44 and GHRH 1-29 peptides whether first 1-29 peptide or full length peptide epitopic regions determine the binding affinity of X-aptamers. To synthesize aptamers against GHRH 1-44 full length target, we used both E.coli HB101 cells to gain high yield of His-GHRH protein. However to compare the binding affinity of x-aptamers against bacterial and eukaryotic GHRH 1-44 peptide, we performed purification from each host. In addition, as HEK293 cells has no expression for GHRH, they express what we transfected through a plasmid and the plasmid has full length GHRH peptide with His-tagged. Thus, HEK293 cells express GHRH 1-44 peptide and this was confirmed by the immunoblotting results.*

**Comment#11 Sequences provided in Figure 2 are aptamers obtained from which selection (1-29 or 1-44)? What is the difference between sequences named TKY2 and TKY?**

**Reply to comment 11:** *The sequences provided in Figure 2 are aptamers obtained from both bacterial/eukaryotic GHRH 1-44 and GHRH 1-29 peptides. Names for TKY2 belongs to bacterial/eukaryotic GHRH 1-44 peptide and TKY belongs to GHRH 1-29 peptide. The detailed explanation was given as “Aptamers against bacterial His-tagged GHRH were TKY2.T1.01, TKY2.T1.02, TKY2.T1.03, TKY2.T1.04, TKY2.T1.05, TKY2.T1.08, TKY2.T1.10, TKY2.T1.12, TKY2.T1.13, TKY2.T1.15, TKY2.T1.17. TKY.T1.01, TKY.T1.02, TKY.T1.03, TKY.T1.04, TKY.T1.05 aptamers were against eukaryotic GHRH NH2 (1-44) peptide. Putative X-aptamers against GHRH 1-29 target named as TKY.T2.02, TKY.T2.06, TKY.T2.07, TKY.T2.08, TKY.T2.09.” in the results section of the revised manuscript.*

**Comment#12 Figure 1 shows expression and purification of NH2 (1-44) target or GHRH NH2 (1-29) target? Figure legends should be more informative, otherwise, the reader has to look back and forth to find the information scattered in the manuscript.**

***Reply to comment 12:*** *We only purified GHRH 1-44 from bacterial and eukaryotic host origin, we bought the GHRH 1-29 peptide from Sigma G6771 as a product. Thus, the figure 1 represents the expression and purification of GHRH 1-44 target. It is mentioned in the figure legend 1 in the revised manuscript.*

**Comment#13. Bacterially expressed His-GHRH protein was expressed as inclusion bodies and denatured using Guanidine. No protocol for protein refolding was presented.**

***Reply to comment 13:*** *Protein refolding by bacterial expressed His-GHRH protein as inclusion bodies and denatured using Guanidine, we gave reference in the revised manuscript. (https://www.ncbi.nlm.nih.gov/pmc/articles/PMC2324003/)*

Review Comments to the Author

Please use the space provided to explain your answers to the questions above. You may also include additional comments for the author, including concerns about dual publication, research ethics, or publication ethics. (Please upload your review as an attachment if it exceeds 20,000 characters)

***Reviewer #1: All comments were resolved and incorporated in the new version of the manuscript. Additional experiments were carried out that clearly dispel the initial doubts of the reviewers***

**Reviewer #2: Authors showed some new data, but the paper remains unacceptable for publication. Besides the authors did not respond satisfactorily to my queries, the manuscript is full of grammar errors, making it almost impossible to understand the messages, which the authors would like to transmit.
Examples for poor English, sometimes together with conceptional errors.**

- *All the grammar errors were corrected, manuscript English is proofread and some sentences were rewritten again in order to transmit the messages.*

**Comment 1: “x-aptamers was used as a detection antibody in order to detect GHRH peptide that applied on GHRH antibody coated plates. “ An aptamer is not an antibody**

***Reply to comment:*** *We changed the sentence as “ According to GHRH ELISA results, instead of using detection antibody, we used x-aptamers to competitively capture GHRH peptide. “ We know that an aptamer is not an antibody but as we dont have any unlabelled aptamers we prefered to use GHRH competitive ELISA in order to demonstrate the specificity of x-aptamers through their binding affinity against target molecule.*

**Comment 2: “To confirm the ELISA results, we performed dot-blot analysis of upper phase of GHRH peptide 296 and X-aptamer combination in dose-dependent manner by dot-blot analysis.**

**Reply to comment 2:** *According to valueable contribution, we changed the sentence as “To confirm the ELISA results, we performed dot-blot analysis in dose-dependent manner“.*

**Comment 3: “As there is not any competitor for GHRH X-aptamers except antibody against GHRH peptide, we performed GHRH ELISA 298 assay. “ Not true, unlabeled aptamers in excess can be used to compete with binding of labeled aptamers.
Reply to comment 3:** *According to valueable contributions, we changed the sentence as “As we do not have any unlabeled aptamers, GHRH antibody is used as a competitor for GHRH X-aptamers against GHRH peptide binding“. We know unlabelled excess aptamer will be used for competitive for GHRH binding to determine specifity of aptamers. However, we synthesized only 5’ biotin labeled aptamers from AM Biochemicals. As we couldn’t purchase both labelled and unlabelled 24 putative aptamers, we can only demonstrate by competitive ELISA Assay in dose-dependent manner.*

**Comment 4: “According to the serum stability results, TKY2.T1.02, TKY2.T1.04, TKY2.T1.05, 304 TKY2.T1.08, TKY2.T1.13, TKY2.T1.17, TKY.T2.05, TKY.T1.05, TKY.T2.02, TKY.T2.07, TKY.T2.08 and TKY.T2.09 X-aptamers were seems to be stable in human serum within 120 h period at 37o “**

**Reply to comment 4:** *We changed the sentence as “According to the serum stability results, TKY2.T1.02, TKY2.T1.04, TKY2.T1.05, TKY2.T1.08, TKY2.T1.13, TKY2.T1.17, TKY.T2.05, TKY.T1.05, TKY.T2.02, TKY.T2.07, TKY.T2.08 and TKY.T2.09 X-aptamers were stable in human serum within 120 h period at 37^o^C (Fig 3e).”*

**Comment 5: “X-aptamers were significantly deplete intracellular cAMP 338 concentration as compared to untreated control cells in HT-29 cells. When we checked the results, no significant effect was observed for cAMP levels for TKY2.T1.08 and TKY2.T1.13 X-aptamers application 340 in HT-29 cells“. Contradictory!**

***Reply to comment 5:*** *The sentence was changed to “Due to cAMP assay analysis, both TKY2.T1.08 and TKY2.T1.13 X-aptamers significantly depleted intracellular cAMP concentration as compared to untreated control cells in HT-29 cells. When we checked the results, no significant effect observed for cAMP levels for TKY2.T1.08 and TKY2.T1.13 X-aptamers application in MIA Paca-2 cells. In addition, we measured statistically significant decline in cAMP levels following TKY.T2.08 and TKY.T2.09 X-aptamers treatment in MIA Paca-2 cells (Fig 6a).”*

Scientific issues:

**Comment 6: PCR cycles: I did not mean SELEX cycles, but PCR cycles needed for amplification of recovered DNA. If i.e. 1 % of the material was recovered following selection, a number of PCR cycles needed to restore 100% can be calculated and certainly will not give 25 cycles.
I know that the technique is patented, but nether the less technical details need to be given, that any researcher can repeat experiments.**

**Reply to comment 6:** *In the method, we do not aim to recover 100% DNA. Instead, the company requested the gel image and informed that it is sufficient for Next Generation Sequencing.*

**Comment 7: Regression plots in Figure 3 are not readable and they seem to rely on a single measurement.**

***Reply to comment 7:*** W*e changed figure as dot-blot analysis and also regression plots in order to increase its readability. The experiment was repeated three times and given figures illustrates the representative of one of the experiment.*

**Comment 8: I asked the authors to provide an alignment of variable sequences, which allows the definition of structural classes. Authors just listed the sequences without any bioinformatical analysis.**

**Reply to comment 8:** *According to comment, we align the variable sequences by bioinformatic analysis and given in the revised version of the manuscript.*

**Comment 9: I asked the authors to adequately introduce X-DNA aptamers (why authors select X-aptamers). This should be done in more detail and not by just citing a reference. The message is confusingly diluted along the paper. However, it should be clearly cited in the introduction.**

**Reply to comment 9:** *We* *explained in detail the X-aptamer technology, advantages and limitations of aptamers. Moreover, we clearly cited why we select X-aptamers in the introduction part.*

***Reviewer #3: The manuscript revision was attached as a word doc file, since it exceeded the limit of 20000 characters***
