# Supplementary material for: Synthesis and characterization of novel ssDNA X-aptamers targeting Growth Hormone Releasing Hormone (GHRH)
Source: PLoS One. 2022 Jan 21;17(1):e0260144. doi: 10.1371/journal.pone.0260144 (PMC8782341; doi:10.1371/journal.pone.0260144)
Supplement: Attachment — Submitted filename: Response to reviewers comments.docx [file pone.0260144.s005.docx]

Response to reviewers comments:

Reviewer #2: I agree that the authors made some progress, but I have yet serious reservations regarding the scientific quality of this manuscript.
***Comment 1. Authors did not understand the point of my concerns of a large number of PCR cycles for reestablishing the X-DNA pool. The point is that over amplification, i.e. by 25 cycles, for reestablishing 100 % from 1 % results in a loss of pool and sequence heterogeneity and selection of artefacts. This is shown in the alignment analysis that authors now provided. It is expected that there are conserved consensus motifs within a part of the previous random region, but not that practically the entire random region makes part of the random regions. If this would be like that, how authors explain the differences in binding affinities.***

***Reply to comment 1:*** Due to valuable comment, we understand that there is a misleading for the selection of aptamers. We didn’t use SELEX for the aptamer selection, we used X-aptamer kit and perform according to manifacturer instructions. The SELEX refers to a different process which itself implies multiple rounds of selection process by using PCR. We did not use SELEX which is a round of PCR amplification. We performed a bead-based aptamer selection. For more information we kindly advise you to watch the process from the following link. (https://www.youtube.com/watch?v=CgfX-KmXdA0). Therefore, we decided to mention the process in the manuscript in a more appropriate way as “We performed a bead-based aptamer selection against two targets, bacterial /eukaryotic GHRH 1-44 and GHRH 1-29 peptide. In addition we replaced “first SELEX” with “first aptamer selection”, and replace “second SELEX” with “second aptamer selection”.  The different cycle numbers of PCR were only to determine the proper cycle number for each fraction to send clean product to sequencing with no overamplification.

***Comment 2. Figure 3c: Where is the control (scrambled sequence aptamer) for these assays. Increase of an unspecific aptamer concentration will augment binding, however cannot be saturated. This needs to be shown. I understand that a non-linear curve using a zero point needs to be performed. Please, check for that TKY2.T1.T2, TKY.T1.01 and TKY.T1.02. The fittings are just wrong. Are these just one-point measurements without any relevance?***

***Reply to comment 2:*** *According to valuable comment, we synthesized scramble sequence aptamer that has similar ΔG value. The sequence was given in the revised manuscript. We performed again dot-blotting with scramble sequence aptamer. Due to new dot-blot results, we analyze the Kd levels again and fit the results at GraphPad prism program. All the new results were given in the revised version of the manuscript. Although it is not a single measurement, we repeat dot-blot via using scramble aptamer.*

***Comment 3. Aptamer alignment by Clustal: So, which is the proposed consensus sequence? Analysis softwares for aptamers are available, including MEME, which are much more suitable than Clustal is.***

***Reply to comment 3:*** *In our previous revision, we performed Cluster alignment program to determine the consensus sequence. However, due to comment we performed MEME analysis for all aptamers instead of Clustal analysis. According to MEME analysis 5’ and 3’ constant sequences and random sequence was given in the revised version of the manuscript.*
